# Somatic genetic rescue of a germline ribosome assembly defect

**DOI:** 10.1038/s41467-021-24999-5

**Published:** 2021-08-19

**Authors:** Shengjiang Tan, Laëtitia Kermasson, Christine Hilcenko, Vasileios Kargas, David Traynor, Ahmed Z. Boukerrou, Norberto Escudero-Urquijo, Alexandre Faille, Alexis Bertrand, Maxim Rossmann, Beatriz Goyenechea, Li Jin, Jonathan Moreil, Olivier Alibeu, Blandine Beaupain, Christine Bôle-Feysot, Stefano Fumagalli, Sophie Kaltenbach, Jean-Alain Martignoles, Cécile Masson, Patrick Nitschké, Mélanie Parisot, Aurore Pouliet, Isabelle Radford-Weiss, Frédéric Tores, Jean-Pierre de Villartay, Mohammed Zarhrate, Ai Ling Koh, Kong Boo Phua, Bruno Reversade, Peter J. Bond, Christine Bellanné-Chantelot, Isabelle Callebaut, François Delhommeau, Jean Donadieu, Alan J. Warren, Patrick Revy

**Affiliations:** 1 Cambridge Institute for Medical Research, Cambridge Biomedical Campus Keith Peters Building, Hills Rd, Cambridge, United Kingdom; 2 Wellcome Trust-Medical Research Council Stem Cell Institute, Jeffrey Cheah Biomedical Centre, Puddicombe Way, Cambridge Biomedical Campus, Cambridge, UK; 3 Department of Haematology, University of Cambridge School of Clinical Medicine, Jeffrey Cheah Biomedical Centre, Puddicombe Way, Cambridge Biomedical Campus, Cambridge, UK; 4 Université de Paris, Imagine Institute, Laboratory of Genome Dynamics in the Immune System, Equipe Labellisée Ligue contre le Cancer, INSERM UMR 1163, Paris, France; 5 INSERM Unité Mixte de Recherche 1163, Structure Fédérative de Recherche Necker INSERM US24/CNRS UMS3633, Genomic Core Facility, Paris Descartes-Sorbonne Paris Cité University, Imagine Institute, Paris, France; 6 French Neutropenia Registry, Assistance Publique-Hôpitaux de Paris, Trousseau Hospital, Paris, France; 7 Institut Necker Enfants Malades, Paris, France; 8 INSERM, U1151, Université Paris Descartes Sorbonne Cité, Paris, France; 9 Université Paris Descartes, Faculté de Médecine Sorbonne Paris Cité, Paris, France; 10 Service de cytogénétique, Hôpital Necker, Assistance Publique-Hôpitaux de Paris, Paris, France; 11 Sorbonne Université, Inserm, Centre de Recherche Saint-Antoine, AP-HP, Hôpital Saint-Antoine, Hématologie Biologique, Paris, France; 12 INSERM Unité Mixte de Recherche 1163, Bioinformatics Platform, Paris Descartes-Sorbonne Paris Cité University, Imagine Institute, Paris, France; 13 Department of Paediatrics, KK Women’s and Children’s Hospital, Singapore, Singapore; 14 SingHealth Duke-NUS Genomic Medicine Centre, Singapore, Singapore; 15 Genome Institute of Singapore, A*STAR, Biopolis, Singapore, Singapore; 16 Bioinformatics Institute (A*STAR), Singapore, Singapore; 17 Department of Biological Sciences, National University of Singapore, Singapore, Singapore; 18 Department of Genetics, Pitié-Salpêtrière Hospital, Sorbonne University, Paris, France; 19 Sorbonne Université, Muséum National d’Histoire Naturelle, UMR CNRS 7590, Institut de Minéralogie, de Physique des Matériaux et de Cosmochimie, IMPMC, Paris, France; 20 Service d’Hémato-Oncologie Pédiatrique, Assistance Publique-Hôpitaux de Paris Hôpital Trousseau, Registre des neutropénies-Centre de référence des neutropénies chroniques, Paris, France; 21 Present Address: PolyProx Therapeutics, Babraham Research Campus, Cambridge, UK; 22 Present Address: MRC Laboratory of Molecular Biology, Francis Crick Avenue, Cambridge Biomedical Campus, Cambridge, UK

**Keywords:** Mutation, Next-generation sequencing, Ribosome, Haematological diseases

## Abstract

Indirect somatic genetic rescue (SGR) of a germline mutation is thought to be rare in inherited Mendelian disorders. Here, we establish that acquired mutations in the *EIF6* gene are a frequent mechanism of SGR in Shwachman-Diamond syndrome (SDS), a leukemia predisposition disorder caused by a germline defect in ribosome assembly. Biallelic mutations in the *SBDS* or *EFL1* genes in SDS impair release of the anti-association factor eIF6 from the 60S ribosomal subunit, a key step in the translational activation of ribosomes. Here, we identify diverse mosaic somatic genetic events (point mutations, interstitial deletion, reciprocal chromosomal translocation) in SDS hematopoietic cells that reduce eIF6 expression or disrupt its interaction with the 60S subunit, thereby conferring a selective advantage over non-modified cells. SDS-related somatic *EIF6* missense mutations that reduce eIF6 dosage or eIF6 binding to the 60S subunit suppress the defects in ribosome assembly and protein synthesis across multiple SBDS-deficient species including yeast, *Dictyostelium* and *Drosophila*. Our data suggest that SGR is a universal phenomenon that may influence the clinical evolution of diverse Mendelian disorders and support eIF6 suppressor mimics as a therapeutic strategy in SDS.

## Abstract (web summary)

Shwachman-Diamond syndrome (SDS) is a leukemia predisposition disorder that is caused by defective release of eIF6 during ribosome assembly. Here the authors show that acquired somatic *EIF6* mutations are frequent in the hematopoietic cells from individuals with SDS and provide a selective advantage over non-modified cells.

## Introduction

In normal individuals, somatic mutations and chromosomal alterations accumulate with age in cells from diverse tissues, including the hematopoietic system^1–9^. The accumulation of spontaneous genetic variations may contribute to age-related disease, organismal aging, and tumorigenesis^10,11^. However, more than 40 years ago, Weill and Reynaud proposed that in certain circumstances, somatic mutations might be beneficial to the cell without inducing disease or cellular transformation^12^. In inherited Mendelian diseases, this phenomenon, dubbed somatic genetic rescue (SGR)^13^, is considered rare and has mainly been observed in hematopoietic disorders, where it may confer a selective advantage and promote recovery of hematopoiesis by counteracting the deleterious effect of the germline mutation^14–16^. In most cases, SGR affects the germline mutated gene (direct SGR^13^). In contrast, indirect SGR involves the acquisition of somatic mutations in a distinct gene that participates in the same pathway that is altered by the germline mutation^13^. For instance, indirect SGR has been highlighted in three independent studies on telomeropathies where somatic promoter-activating mutations in *TERT*, the gene encoding the telomerase catalytic subunit that elongates telomeres, were identified in blood cells from patients with germline mutations in genes involved in telomere length regulation, i.e., *TERT*, *TERC*, *PARN*, and *NHP2*^17–19^. To the best of our knowledge, indirect SGR has only been described to date in the telomeropathies.

Shwachman-Diamond syndrome (SDS; OMIM #260400) is a rare autosomal recessive disease characterized by bone marrow failure, poor growth, skeletal defects, exocrine pancreatic insufficiency, and predisposition to hematological malignancies^20^. Biallelic mutations in *SBDS* are the predominant cause of SDS, but biallelic *EFL1* mutations have also been identified^21–23^. SBDS and the GTPase EFL1 cooperate to evict the anti-association factor eIF6 (yeast Tif6) from the nascent large ribosomal subunit^23–25^, an essential prerequisite that allows the 60S and 40S subunits to join to form mature, actively translating 80S ribosomes^26^. Hence SBDS and EFL1 deficiencies are considered as ribosomopathies since they lead to impaired ribosomal subunit joining and reduced protein synthesis as a consequence of defective eIF6 eviction from the 60S subunit^20,23–25,27,28^.

Recurrent mosaic acquired interstitial deletions of chromosome 20 (del(20q)) encompassing the *EIF6* gene have been detected in bone marrow cells from some individuals with SDS^29–31^. This observation led to the proposal that a reduced dose of eIF6 due to del(20q) might be advantageous to SDS cells by bypassing the defect in ribosomal subunit joining, representing a novel mechanism of indirect SGR^13,29–31^. However, the minimal del(20q) region characterized in hematopoietic cells in SDS spanned 2.2 Mb, encompassing 28 genes in addition to *EIF6*^31^. Furthermore, del(20q) is one of the most common mosaic chromosomal alterations associated with age-related clonal hematopoiesis^7–9^. Thus, it remains unclear whether *EIF6* haploinsufficiency generated by del(20q) indeed represents a *bona fide* mechanism of indirect SGR in SDS hematopoietic cells.

Here, we test the hypothesis that acquired somatic mutations in the *EIF6* gene might provide a selective advantage for hematopoietic cells in SDS that promotes their clonal expansion. We performed ultra-deep sequencing of the *EIF6* gene in hematopoietic cells from 40 individuals with SDS carrying biallelic germline *SBDS* mutations, identifying mosaic somatic *EIF6* mutations in 60% of SDS patients but not in healthy donors. By combining functional studies in yeast, *Dictyostelium discoideum*, and *Drosophila melanogaster* with structural analysis and molecular dynamics (MD) simulations, we show that acquired somatic *EIF6* missense mutations that reduce eIF6 dosage or eIF6 binding to the 60S subunit bypass SBDS deficiency by rescuing the defects in ribosome assembly and global protein synthesis. Our results establish that the acquisition of somatic *EIF6* mutations is a frequent mechanism of indirect somatic genetic rescue in hematopoietic cells in SDS, suggesting a strategy for the development of disease-modifying targeted therapeutics in SDS.

## Results

### *EIF6* mutations as a mechanism of somatic genetic rescue in SDS

**Fig. 1:**
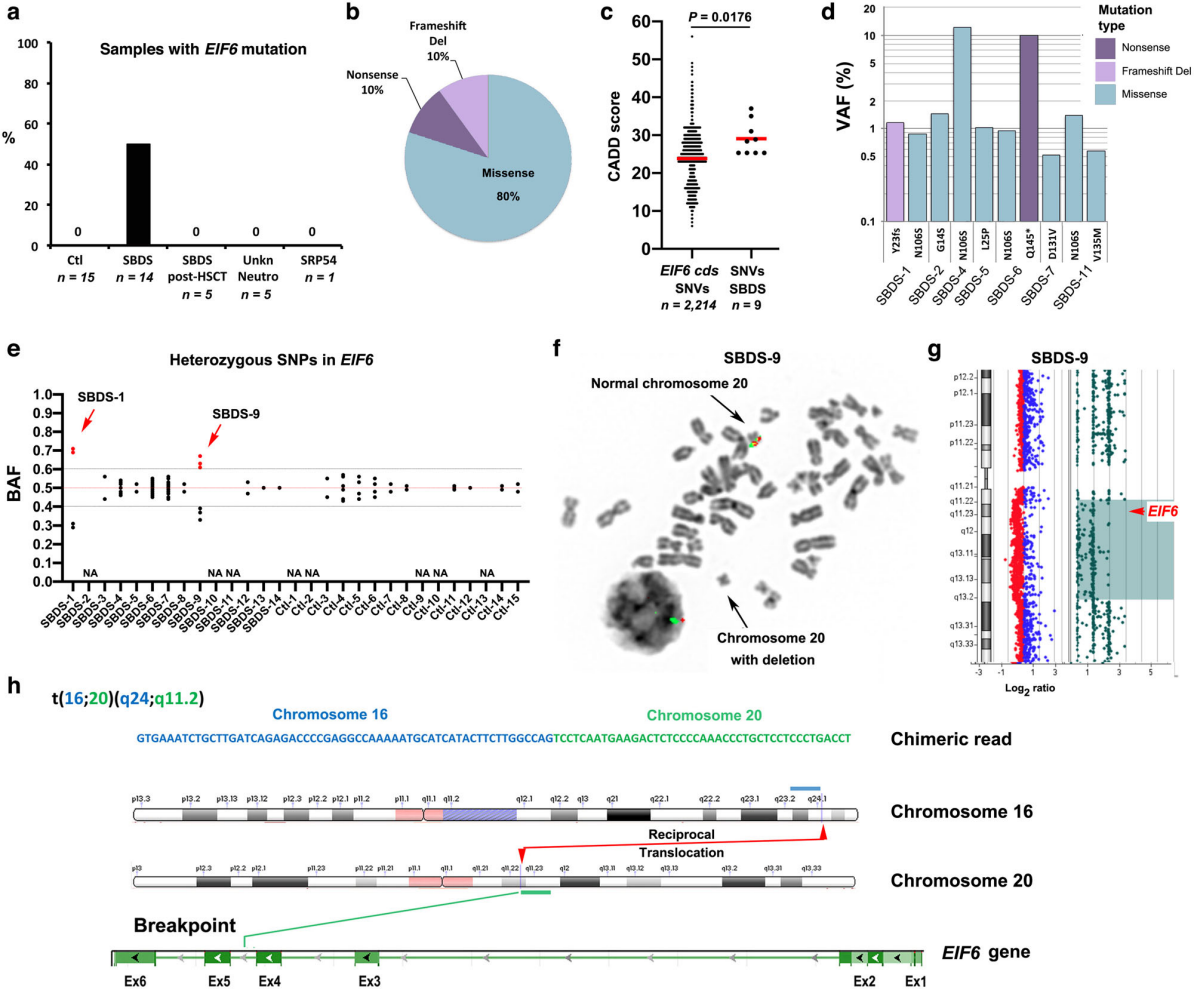
Multiple somatic genetic events target the *EIF6* gene in hematopoietic cells in SDS. **a** Somatic *EIF6* mutations are common in SDS. The percentage of individuals with *EIF6* mutations in the specific groups of patients is indicated. **b** Classification of identified *EIF6* mutations. **c** CADD scores of all the possible SNVs in the coding sequence of *EIF6* (*n* = 2214; Supplementary Data 2) versus the 9 SNVs in *EIF6* identified in SDS patients. Red bars correspond to mean values. A two-tailed *p*-value of the unpaired t-test is indicated. **d** VAF of the 10 identified *EIF6* mutations identified in the indicated SDS patients. **e** BAF of the heterozygous single nucleotide polymorphisms (SNPs) located in *EIF6* in SDS patients and healthy controls. NA: not available. **f** Detection of interstitial del(20q) by metaphase cytogenetics with fluorescent probes located 7 Mb downstream of the *EIF6* gene in bone marrow cells from patient SBDS-9 (Supplementary Fig. 1). **g** Large heterozygous mosaic genomic deletion on chromosome 20 encompassing the *EIF6* gene (red arrow) detected by array comparative genomic hybridization (CGH) in bone marrow cells from patient SBDS-9. **h** Identification of the breakpoint in the reciprocal translocation t(16; 20)(q24; q12) within intron 4–5 of *EIF6* on chromosome 20q. Chromosome 16 sequence is blue, chromosome 20 is green.

To determine whether acquired mutations in *EIF6* represent a mechanism of SGR in hematopoietic cells in SDS, we performed ultra-deep targeted sequencing of the full genomic *EIF6* gene (introns/exons) after hybridization-based capture with biotinylated ssDNA probes designed and prepared to target a 123 kb chromosomal locus encompassing *EIF6* (chr20:35,256,992-35,380,631 according to the GRCh38.p12 assembly of the human reference genome). We analyzed a total of 14 SDS patients (hereafter denoted SBDS) carrying biallelic germline mutations in the *SBDS* gene (mean age: 14.7 years; range 1–38.2; DNA extracted from blood: *n* = 8; DNA extracted from bone marrow: *n* = 6; Supplementary Data 1). We also tested 5 SDS patients who had undergone hematopoietic stem cell transplantation (denoted SBDS post-HSCT; DNA extracted from blood) and fully reconstituted their hematopoietic system as inferred by wild type (WT) *SBDS* sequence in peripheral blood cells (100% donor). In addition, we tested 5 patients with neutropenia of uncharacterized genetic origin (denoted Neutro Unkn; in 4, DNA was extracted from blood, in 1 from bone marrow), one SDS-like patient carrying biallelic *SRP54* mutations^32^ (denoted SRP54; DNA from blood), and 15 healthy age-matched donors (denoted Ctl, DNA from blood). After removing duplicates, ultra-deep *EIF6* sequencing provided a mean depth of 2807X (ranging from 718X to 7940X). To accurately identify *EIF6* genetic variants with low rates of somatic mosaicism, we considered all detected genetic variants in the *EIF6* coding sequence with variant allele frequencies (VAF) ≥0.5% as somatic *EIF6* mutations. Using this criterion, we did not detect *EIF6* mutations in the 15 healthy controls, the 5 SDS patients post-HSCT, the 5 patients with neutropenia of unknown molecular origin, or the SRP54-deficient patient. In contrast, we detected a total of 10 *EIF6* mutations in 7 of the 14 SDS patients (50%) (Fig. 1a). Nine mutations corresponded to single nucleotide variation (SNVs; 8 missense and 1 nonsense), while one was a 5 bp deletion predicted to cause a frameshift and a premature stop codon (Fig. 1b). The combined annotation-dependent depletion (CADD) score represents a predictive indicator of the deleterious effect of a genetic variant^33^. Noticeably, the mean CADD score for the 9 *EIF6* SNVs identified in SDS patients was significantly higher than the mean CADD score generated by all possible SNVs in the *EIF6* coding sequence (synonymous, missense, nonsense, start/stop-loss; Fig. 1c and Supplementary Data 2). This observation suggests that clones carrying *EIF6* SNVs predicted to have a high deleterious impact were preferentially amplified in blood cells from SDS patients. Moreover, the absence of somatic *EIF6* mutations in normal individuals suggests that they are not favored in cells in normal conditions.

The mean VAF for the 10 *EIF6* mutations was 2.15% (range 0.51–12.32%). In 3 SDS patients, we detected 2 different *EIF6* mutations (Fig. 1d and Supplementary Data 1), indicating that distinct *EIF6* mutated clones can emerge independently within the same individual. Strikingly, the same somatic mutation (g.20:33868509A>G; c.317A>G) leading to the eIF6 substitution N106S was detected in four unrelated SDS patients with a VAF ranging from 0.87 to 12.32%. This suggested to us that N106S might represent a recurrent somatic mutation with a key functional impact in SBDS deficient cells (see below) (Fig. 1d and Supplementary Data 1).

We next analyzed the B-allele frequency (BAF) across all heterozygous single nucleotide polymorphisms (SNPs) located in the *EIF6* gene. In 9 SDS patients and 10 healthy individuals in whom SNPs were informative, the BAFs were around 0.5, as expected for heterozygous SNPs in diploid cells^34^. In contrast, two SDS patients (SBDS-1 and SBDS-9) exhibited a sharp BAF deviation from 0.5 (Fig. 1e and Supplementary Data 3), suggesting the existence of a mosaic genetic deletion encompassing the *EIF6* gene. The combination of cytogenetic analysis using specific FISH probes located near the *EIF6* locus (Supplementary Fig. 1) and array comparative genomic hybridization (CGH) confirmed the presence of an interstitial 20q11.21-q13.2 deletion encompassing *EIF6* in a bone marrow sample from patient SBDS-9 that was estimated to affect 37% of cells (Fig. 1f, g, and Supplementary Data 1).

Although ultra-deep *EIF6* sequencing did not detect *EIF6* mutations in bone marrow cells from patient SBDS-3, the cytogenetic analysis highlighted a reciprocal translocation t(16;20)(q24;q11.2) in 2 out of 20 metaphases (Supplementary Data 1). Since the *EIF6* gene maps to 20q11.2, we wondered whether the breakpoint in chromosome 20 was located within the *EIF6* gene. A search for chimeric reads from the ultra-deep sequencing containing both the *EIF6* gene and chromosome 16 sequences unveiled chimeric sequences in patient SBDS-3 but not in 4 controls. Analysis of chimeric reads precisely positioned the translocation breakpoints in chromosome 20 within intron 4–5 of *EIF6* and in a non-coding region of chromosome 16 between the *COX4* (9175 bp at 5’ side) and the *IRF8* genes (86,642 bp at 3’ side) (Fig. 1h). We conclude from this analysis that the translocation t(16;20)(q24;q11.2) detected in a mosaic state in bone marrow cells from patient SBDS-3 disrupted one copy of *EIF6* to cause haploinsufficiency.

We conclude that multiple distinct somatic genetic events affecting the *EIF6* gene are frequent in hematopoietic cells in SDS but not in healthy individuals. These de novo mosaic genetic modifications consist of chromosomal alterations affecting *EIF6* (interstitial del(20q), reciprocal translocation) or somatic point mutations in the *EIF6* coding sequence (nonsense, missense, and small deletions). These findings support our hypothesis that *EIF6* mutations indeed represent a mechanism of indirect SGR that promotes clonal expansion in the context of a germline ribosome assembly defect in SDS.

### The spectrum of acquired somatic *EIF6* mutations in SDS

**Fig. 2:**
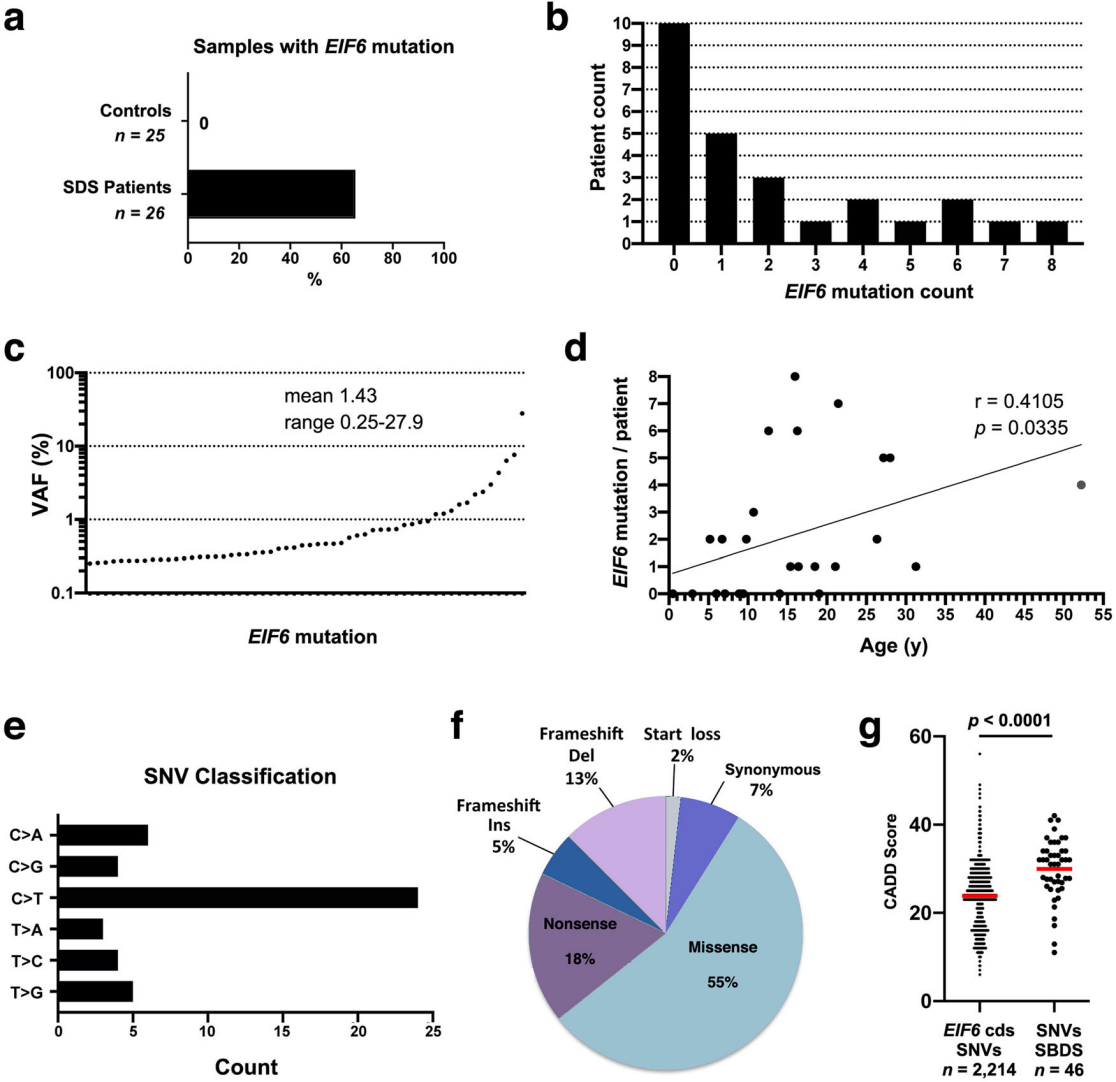
Somatic *EIF6* mutations identified in SDS. **a** Percentage of SDS patients carrying somatic *EIF6* mutations. **b** *EIF6* mutation count across the 26 SDS patients. **c** VAF distribution of the 56 identified *EIF6* mutations detected by ultra-deep sequencing. **d** Mutation count in each individual versus age. **e** Mutational spectrum of the 46 SNVs identified in *EIF6*. P-value and Pearson correlation are indicated. **f** Classification of the 56 mutations identified in *EIF6*. **g** CADD scores of all the possible SNVs (*n* = 2214; Supplementary Data 2) in *EIF6* coding sequences versus the CADD scores of the 46 SNVs identified in the SDS patients. Red bars correspond to mean values. A two-tailed *p*-value of the unpaired t-test is indicated.

To strengthen this initial genetic analysis, we performed ultra-deep *EIF6* sequencing of a larger cohort consisting of 26 SDS patients carrying biallelic *SBDS* mutations (mean age: 15.4 years, range 0.47–52.2 years; DNA from blood cells: *n* = 3; DNA from bone marrow: *n* = 23, Supplementary Data 1) and 25 age-matched healthy individuals (DNA from blood cells: *n* = 25). To increase the depth of sequencing with a limited quantity of DNA, we modified the hybridization-based capture strategy by using the *EIF6* cDNA (1016 bp) as sequence bait. After duplicate removal, this approach yielded a mean depth of 26,873X (range 11,140–47,185X). In this setting, we considered all genetic variants in the *EIF6* coding sequence with a VAF of ≥0.25% as somatic *EIF6* mutations. In total, we identified 56 *EIF6* mutations in 17 of the 26 SDS patients (65.3%), but none in the 25 healthy donors (Fig. 2a). Up to 8 different *EIF6* mutations were present in the same individual (mean 2.07; range 0–8) (Fig. 2b). The mean VAF in patients carrying *EIF6* mutations was 1.43% (range 0.25–27.9%) (Fig. 2c). Congruent with the reported accumulation of somatic mutations in hematopoietic cells over time^5,6^, we found a slight but significant positive linear correlation between the *EIF6* mutation count and age (*r* = 0.4105; *p* = 0.0335; Pearson correlation) (Fig. 2d). However, the cumulative VAF per patient among SDS patients carrying *EIF6* mutations did not correlate with age or mutation count (*r* = 0.04629; *p* = 0.86 and *r* = 0.03589; *p* = 0.8912, respectively, Supplementary Fig. 2). Among the 56 *EIF6* mutations, 46 were SNVs (82.1%) that mainly consisted of C>T transitions (51.1%), a mutational spectrum that likely reflects the spontaneous deamination of cytosine residues observed in hematopoietic cells from normal individuals^5,6,35^ (Fig. 2e). Thirty-one were nucleotide substitutions leading to missense mutations (55.3%), 20 corresponded to nonsense or small indels inducing frameshift and premature stop codons (35.7%), 4 were synonymous (7.1%) and one corresponded to loss of the start codon (1.8%; M1L) (Fig. 2f). The mean CADD score of these 56 SNVs was significantly higher than the mean CADD scores of all possible *EIF6* SNVs (Fig. 2g). Furthermore, the mutation spectrum among the SNVs highlighted 3.4 fold more non-synonymous mutations than expected neutrally, as inferred by the ratio of non-synonymous to synonymous variants (dN/dS = 3.4; with dN/dS = 1 representing neutrality)^36^. Together, these results further argue that *EIF6* mutations predicted to have a functional impact are positively selected in hematopoietic cells in SDS. Of note, the interrogation of gnomAD, COSMIC, and TCGA databases indicated that these mutations were absent or only present at a very low frequency in normal individuals and tumors (Supplementary Table S1).

**Fig. 3:**
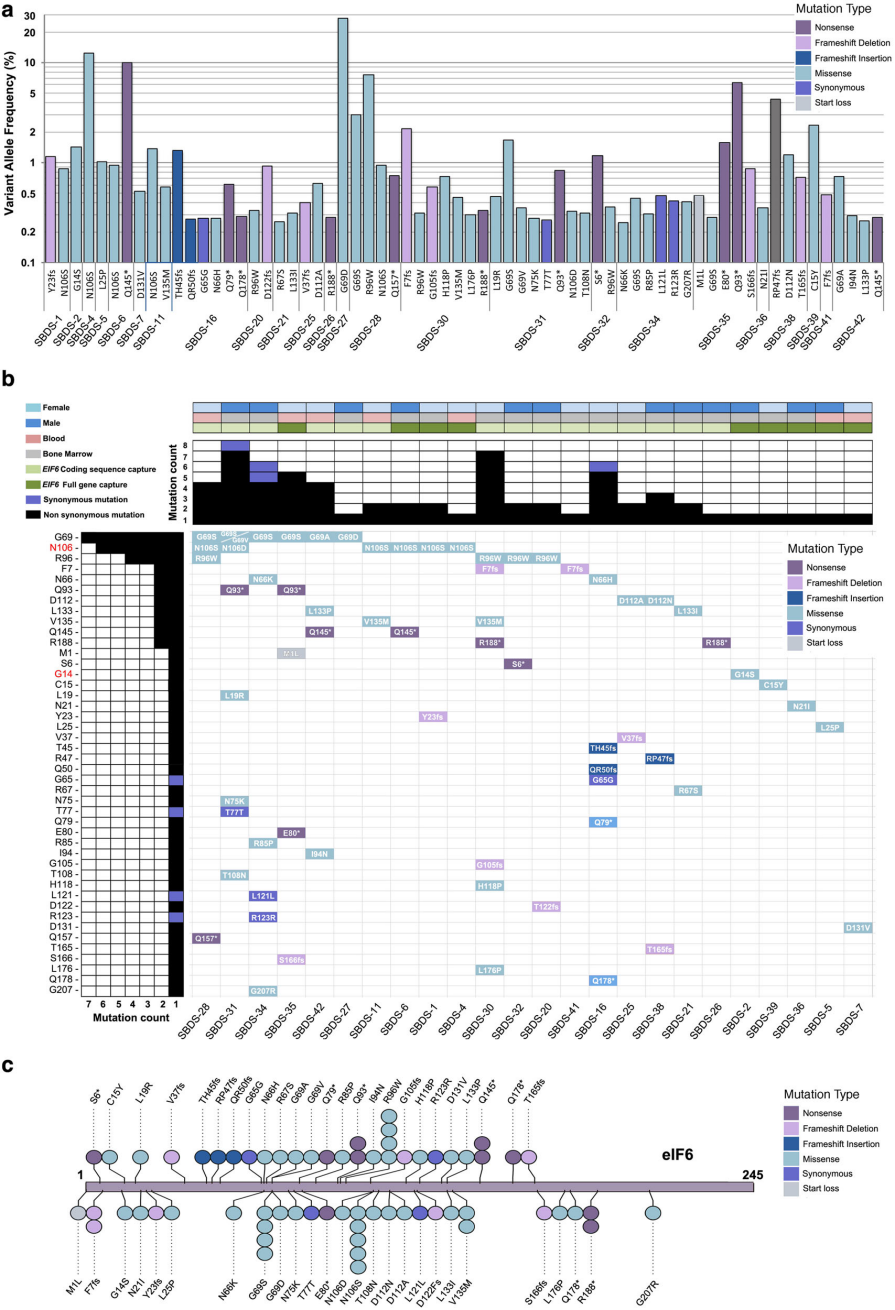
Spectrum of somatic *EIF6* mutations in SDS hematopoietic cells. **a** Spectrum of 66 mutations and their corresponding VAFs identified by ultra-deep sequencing in 24 SDS patients. **b** Waterfall plot of the 66 mutations highlighting the recurrently impacted residues. N106S and G14S (highlighted in red on the left) represent gain-of-function mutations identified in Sdo1-deleted yeast cells^25^. Gender of patients, the origin of DNA, and the method of *EIF6* capture for deep-sequencing are indicated. Purple cases represent synonymous mutations. Colors denote the type of mutation as listed in the inset (upper right corner). **c** Lolliplot showing the distribution of mutations in eIF6.

Collectively, from two independent genetic analyses, we identified a total of 66 somatic eIF6 mutations in 24 out of 40 SDS patients (60%) of which 54 (81.8%) are missense mutations (Fig. 3a, b) that are distributed throughout the protein (Fig. 3c). Five SDS patients (12.5%) exhibited clones with a VAF higher than 5%. The clones with a VAF >5% harbored either nonsense (Q93*, VAF = 6.34%; Q145*, VAF = 10%) or missense *EIF6* mutations (G69D, VAF = 27.9%; R96W, VAF = 7.59%; N106S, VAF = 12.32%) and 19 SDS patients (47.5%) exhibited a cumulative VAF >1% (Fig. 3a and Supplementary Data 1). Strikingly, 7 amino acids (N66, G69, R96, N106, D112, L133, and V135) were recurrently targeted by missense mutations (Fig. 3a, b and Supplementary Data 1): 6 patients carried 7 SNVs affecting residue G69, generating distinct missense substitutions (G69A; G69S; G69V; G69D) (Fig. 3b); 4 patients carried the same R96W substitution; 4 patients carried mutations affecting residue N106 (N106S; N106D), 2 patients had mutations affecting residue N66 (N66H; N66K); 2 patients harbored mutations affecting residue D112 (D112N; D112A); 2 patients carried mutations affecting residue L133 (L133P; L133I) and 2 patients harbored the same V135M mutation (Fig. 3b). Noteworthy, among the somatic missense mutations revealed, G14S and N106S (Fig. 3b) were previously identified as suppressor mutations that bypassed the ribosome assembly defect in yeast cells lacking the SBDS homolog, Sdo1^25^. These findings further support the notion that our ultra-deep sequencing had identified mutations that drive positive clonal selection in the context of human SBDS deficiency in vivo, likely by increasing fitness at the cellular level.

There was no statistical correlation between the presence of *EIF6* mutations (or their VAF) and hemoglobin, platelet, or white cell count in SDS individuals at the time of DNA sampling for *EIF6* sequencing (Supplementary Fig. 3 and Supplementary Data 1).

In sum, our genetic analysis demonstrates that clones carrying somatic genetic mutations in the *EIF6* gene are frequent in blood and bone marrow cells from SDS patients, suggesting that they provide a cellular selective advantage in this context. Some of these events, *i.e*. interstitial deletion, reciprocal translocation, nonsense, and small indels are predicted to generate *EIF6* null alleles, provoking *EIF6* haploinsufficiency. Next, we set out to assess the impact of these mutations by structural, biochemical, and functional analysis.

### Three categories of recurrent missense mutations in eIF6

**Fig. 4:**
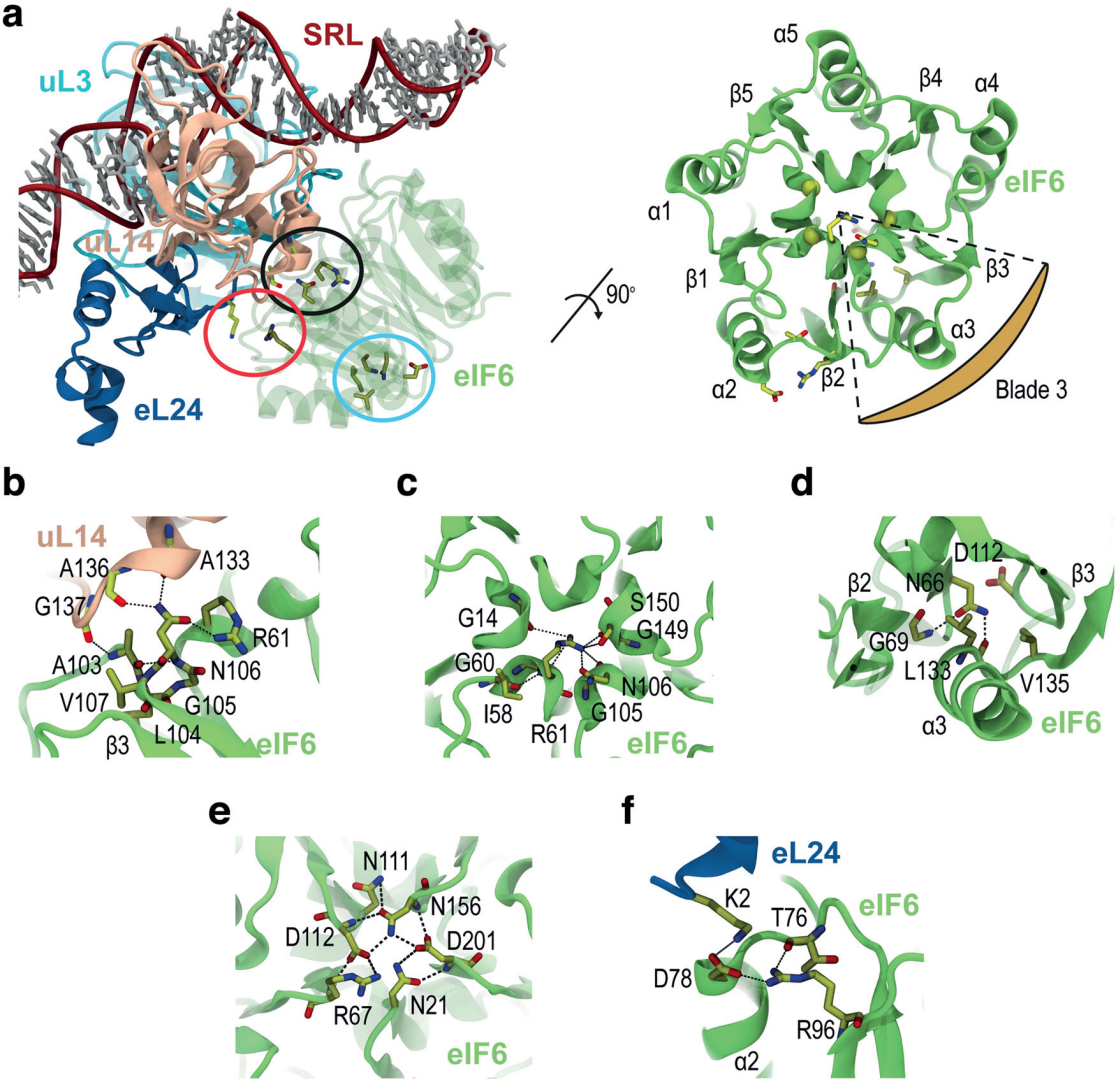
SDS-related eIF6 mutations map to three regions. **a** Atomic model (two orthogonal views) of the interface between human eIF6 and the 60S ribosomal subunit (based on PDBID 7OW7). The eIF6 residues mutated in the SDS cluster in three independent regions highlighted in black (interface with uL14), cyan (interface between blades 2 and 3), and red (eL24 interface) ellipses. **b**–**f** Stabilizing interactions formed by SDS-related eIF6 residues N106 (**b**), R61 (**c**), N66, G69, L133, V135 (**d**), D112 (**e**), and R96 (**f**). eL24 is blue; uL14, salmon; eIF6, green. SRL, sarcin-ricin loop. Figures were generated using VMD (see “Methods” section).

We focused on the eIF6 amino acids (N66, G69, R96, N106, D112, L133, and V135) that are recurrently targeted in SDS. These residues are highly conserved across species, with 5 out of the 7 amino acids conserved from *Homo sapiens* to the archaeon *Methanopyrus kandleri* (Supplementary Fig. 4). We used the 2.4 Å cryo-EM structure of human eIF6 bound to the human 60S subunit (PDBID: 7OW7) to map the eIF6 mutations (Fig. 4a). As first described for the two homologs in *Methanocaldococcus jannaschii* and *Saccharomyces cerevisiae*^37^, eIF6 has a pentein fold consisting of five repeated subunits, with 3-stranded ß-sheets arranged as blades around a five-fold axis of pseudo-symmetry (Fig. 4a). The radial arrangement of these subunits is closed by a “velcro” strategy, with the last ß-strand of the last blade provided by the N-terminal ß-strand, as in ß-propeller 3D structures. Five small helices form an inner ring that includes a position invariably occupied by a small amino acid residue (G, A) to allow tight packing (Fig. 4a and Supplementary Fig. 4). Both sides of the pentein fold form flat surfaces, one of which forms the interface with ribosomal proteins uL14 (RPL23), eL24 (RPL24), uL3 (RPL3) (using the new nomenclature^38^), and the sarcin-ricin loop (SRL) (Fig. 4a). We mapped the seven recurrently mutated amino acids to three regions of the eIF6 protein. The first (highlighted in black in Fig. 4a) includes residue N106 (blade 3) which is mutated (N106S and N106D) in 6 SDS individuals (Fig. 3b). The side chain of N106 forms hydrogen (H)-bonds with the main chain oxygen atoms of uL14 residues A133 and A136 (Fig. 4b). In addition, the backbone nitrogen of N106 forms an intra-molecular H-bond with the backbone oxygen of residue A103. In turn, the backbone nitrogen of A103 forms an H-bond with the backbone oxygen of uL14 residue G137. The side-chain and backbone atoms of N106 also form intra-protein H-bonds with the side-chain and backbone atoms of R61 (blade 2) (Fig. 4b). A network of H-bonding interactions links R61 (blade 2) with the main chain oxygen atoms of G14 (blade 1), I58, G60 (blade 2), and G149 (blade 4) (Fig. 4c). Interestingly, an R61L mutation was recently identified in a patient with a clinical phenotype consistent with SDS^39^. The second region (highlighted in cyan in Fig. 4a) contains 5 amino acids that cluster at the interface between blade 2 (N66 and G69) and blade 3 (D112, L133, and V135) (Fig. 4d). Residue N66 forms H-bonds with the main chain oxygen atoms of G69 and L133, while the side chains of L133 and V135 form hydrophobic interactions. At the solvent-exposed core of eIF6, D112 forms H-bonds with the backbone nitrogen of R67 and the side chain of N156 (blade 4) as part of a wider network of H-bonds involving residues N21 (blade 1), N111 (blade 3), and D201 (blade 5) (Fig. 4e). Mutation of any of the five residues lying within the second hotspot is predicted to destabilize the pentein fold as a whole. The third region (highlighted in red in Fig. 4a) contains residue R96 (at the end of strand ß3 of blade 2), which forms an intra-protein H-bond with the backbone of residue T76 (blade 2) (Fig. 4f). This interaction may help promote polar interactions between eIF6 residue D78 (blade 2) and eL24 residue K2. The recurrent R96W mutation, identified in 4 SDS patients, likely disrupts both the stability of blade 2 and the interaction of eIF6 with eL24.

### *EIF6* mutations rescue fitness defects of SBDS-deficient cells in vivo

**Fig. 5:**
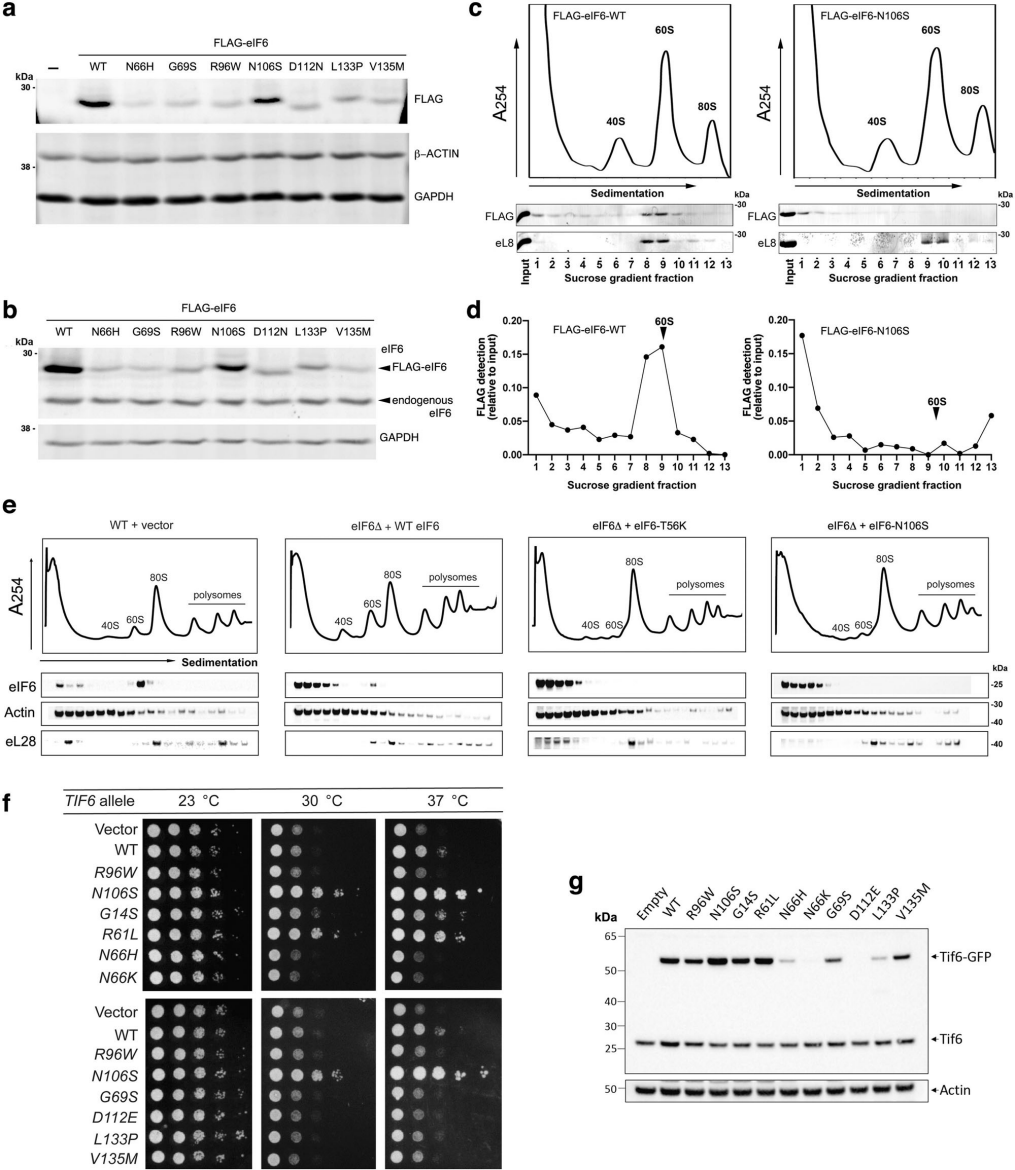
Functional consequences of SDS-related eIF6 mutations. **a**, **b** The eIF6-N106S mutation does not alter eIF6 protein stability in human cells. Cell extracts from HEK293T cells were immunoblotted to detect the indicated FLAG-eIF6 variants compared with (**a**) GAPDH, β-ACTIN, or (**b**) endogenous eIF6. Representative of three independent experiments. **c** The N106S mutation reduces eIF6 affinity for the 60S subunit in human cells. Cell extracts from HEK293T cells transfected with FLAG-eIF6-WT or FLAG-eIF6-N106S were fractionated by sucrose gradient sedimentation and immunoblotted to visualize eIF6 or eL8. Representative of two independent experiments. **d** Quantification of FLAG-eIF6 expression in the experiments depicted in **c**. **e** The eIF6-N106S and eIF6-T56K mutants have a lower affinity for the 60S subunit in *Dictyostelium* cells. Extracts from eIF6-deleted (*EIF6Δ*) *Dictyostelium* Ax2 cells transformed with plasmids expressing eIF6-T56K or eIF6-N106S variants versus WT cells transformed with vector alone were fractionated by sucrose gradient sedimentation and immunoblotted to visualize the indicated proteins (3 replicates). **f** SDS-related Tif6 missense variants rescue the fitness defect of Sdo1-deficient cells. Tenfold serial dilutions (from left to right) of conditional Sdo1-deficient (*sdo1*^*ts*^) cells complemented with plasmids expressing empty vector (pRS316), WT Tif6 or the indicated Tif6 variants were spotted onto SD-URA medium at the permissive (23 °C, 3 days) or restrictive (30 °C, 2 days; 37 °C, 3 days) temperatures. **g** SDS-related Tif6 missense mutations that map to the uL14-binding interface do not alter protein stability. Cell extracts from *sdo1*^*ts*^ cells expressing empty vector, WT, or mutant Tif6-GFP were immunoblotted to detect Tif6 or actin loading control (3 replicates).

We next set out to test the impact of the N66H, G69S, R96W, N106S, D112N, L133P, and V135M mutations on eIF6 protein expression, stability, and function. Immunoblotting of extracts from HEK293T cells transfected with equal amounts of WT and mutant FLAG-tagged eIF6-expressing vectors indicated that all but the N106S mutation reduced eIF6 expression, consistent with a reduction in eIF6 stability as predicted by the structural analysis (Fig. 5a and Supplementary Fig 5). We further verified that the ectopic expression of the FLAG-eIF6 mutants did not affect the expression and/or stability of the endogenous eIF6 protein (Fig. 5b). These observations suggest that the selective advantage provided by the N106S mutation is not due to reduced eIF6 dosage, in contrast to the N66H, G69S, R96W, D112N, L133P, and V135M variants (Fig. 5a, b).

We assessed the ability of the eIF6 N106S mutant to interact with the 60S subunit. Immunoblots of sucrose gradient fractions from HEK293T cells transfected with vectors expressing either WT FLAG-eIF6 or N106S proteins indicated that, unlike WT FLAG-eIF6, the N106S mutant did not co-sediment with the 60S subunit (Fig. 5c, d). We next examined the distribution of WT eIF6 versus the mutants T56K (the most potent gain-of-function mutation identified in yeast^25^) and N106S when expressed in *Dictyostelium discoideum* Ax2 cells lacking the endogenous *EIF6* allele by sucrose gradient fractionation and immunoblotting of cell extracts. Both the endogenous and over-expressed WT eIF6 but not the eIF6-T56K or N106S variants, co-fractionated with the 60S subunit (Fig. 5e). Furthermore, WT eIF6 but not the T56K or N106S variants, induced a functional defect in ribosomal subunit joining in Ax2 cells (Fig. 5e).

We next tested the ability of SDS-associated eIF6 missense mutations to rescue the fitness defect of SBDS-deficient cells in vivo by engineering a conditional mutation in the yeast SBDS homolog Sdo1 (*sdo1*^*ts*^), based on a temperature-sensitive intein which is spliced out to create a functional Sdo1 protein at the permissive (23 °C) but not the restrictive temperatures (30 °C or 37 °C)^28,40^. Compared with empty vector or WT Tif6 controls, expression of the Tif6-G14S, R61L, and N106S mutants (but not N66H, N66K, G69S, R96W, D112E, L133P, and V135M), rescued the fitness defect of *sdo1*^*ts*^ cells at the restrictive temperatures (Fig. 5f). Immunoblotting revealed that cofractionation of the Tif6-R61L variant with the 60S subunit was reduced compared to endogenous WT Tif6 (Supplementary Fig. 6) and that all but the G14S, R61L and N106S mutations decreased Tif6 expression relative to the endogenous Tif6 protein (Fig. 5g). These data confirm that SDS-related Tif6 missense mutations that map to the interface with uL14 act as dominant gain-of-function mutations that are able to bypass the fitness defect caused by Sdo1 deficiency and suggest that mutations that destabilize the Tif6 protein confer loss-of-function. We validated this hypothesis by showing that the mutants with the most marked reduction in protein expression (Tif6-N66H, N66K, and D112E) failed to rescue a *tif6Δ* allele in haploid yeast cells (Supplementary Fig. 7), thereby identifying these as *bona fide tif6* null alleles. Given the conservation of eIF6 function from human to prokaryotes, collectively these observations strongly support the hypothesis that in SDS, hematopoietic cells positively select somatic mutations that either impair the interaction of eIF6 with the 60S subunit, reduce the level of eIF6 expression or indeed completely abrogate eIF6 function.

### N106S mutation dynamically disrupts the H-bonding interface between eIF6 and uL14

**Fig. 6:**
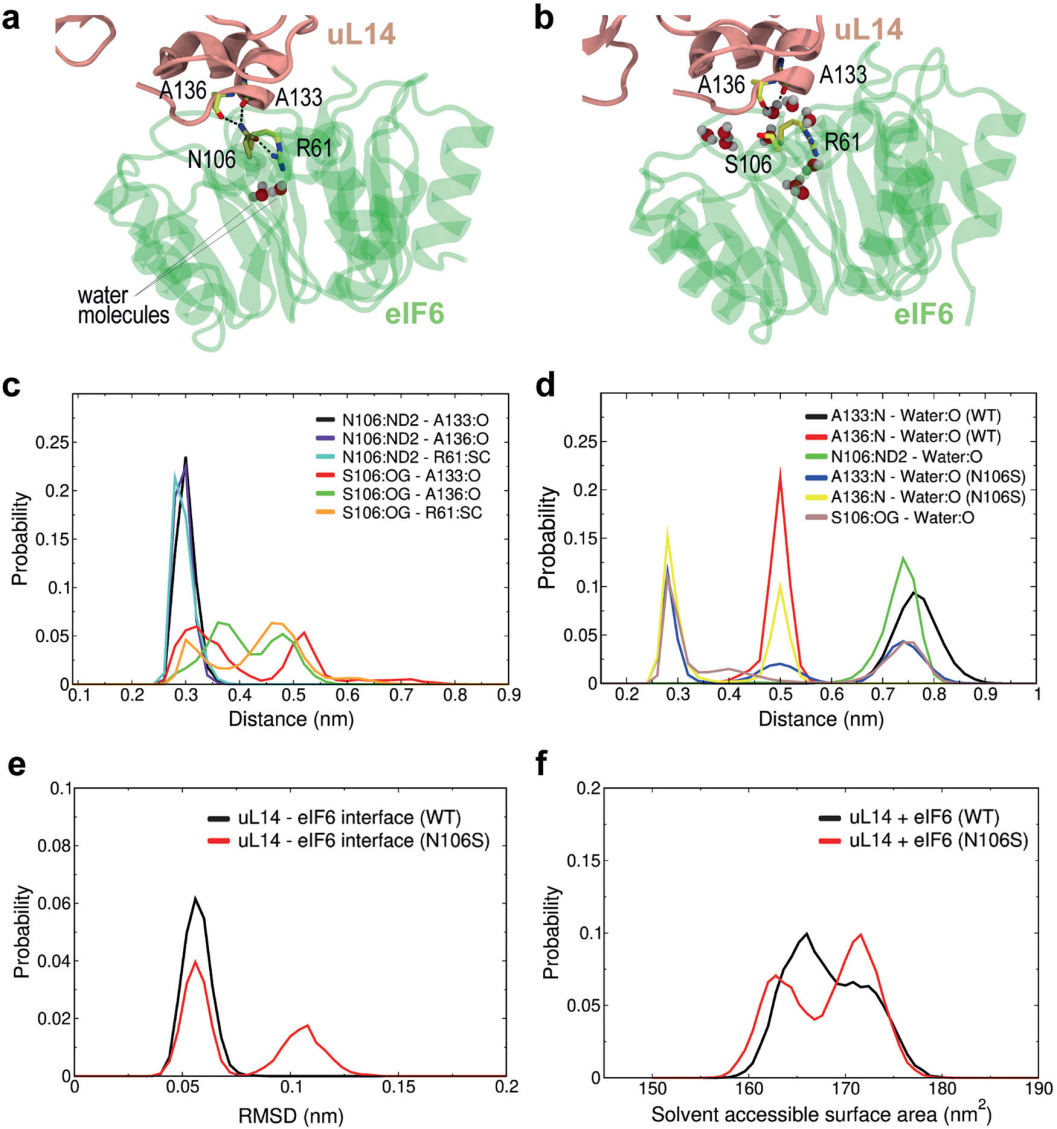
N106S mutation disrupts the H-bonding capacity of the eIF6-uL14 interaction interface. **a**, **b** Representative snapshots of the interaction interface between eIF6 N106 WT or S106 mutant (green) and uL14 (salmon) after 500 ns of simulation. Key water molecules are indicated in CPK format. **c**, **d** Distances (nm) between the indicated atoms of eIF6 WT and mutant (residues N106, S106, and R61), and either uL14 (residues A133, A136) (**c**) or water (**d**). **e** Root mean square deviation (RMSD) of the distance (nm) between the WT or mutant eIF6 inner ring and uL14. **f** Solvent accessible surface area of the WT or mutant eIF6-uL14 complex. Curves in each plot include data from 5 replicas. “SC”, sidechain atoms NH1 and NH2 of the R61 guanidinium moiety.

To provide additional insights into the mechanism by which the recurrent SDS-related eIF6 missense mutation N106S destabilizes the interaction interface with uL14, we utilized atomic-resolution MD simulations to study the stability of a solvated complex comprising eIF6, uL14, eL24, uL3, and a double-stranded helical segment of the 28S ribosomal RNA. Five 500 ns replica simulations were performed for both the WT system and the in silico eIF6 N106S mutant (Fig. 6). In the WT simulations, the N106 side chain maintained stable H-bond contacts with the backbone carbonyls of uL14 residues A133 and A136, with an average donor-acceptor distance of 2.9 Å (Fig. 6a, c, and Supplementary Fig. 8a–j). The sidechain amide oxygen atom in N106 also retained its native intramolecular contacts with R61 (Fig. 6c and Supplementary Fig. 8a–j), bridging uL14 with the internal network of eIF6 H-bonding interactions spanning blades 1–5, as described above. Thus, simulations of the WT complex demonstrated that the key contacts observed in the cryo-EM structure were largely reproduced (Fig. 4b). By contrast, a similar analysis of the eIF6 mutant revealed significant destabilization around S106 in 3 out of 5 replicas (Supplementary Fig. 8b, d, e, g, i, j). The serine sidechain hydroxyl was only able to form weak, intermittent H-bonds with the backbone carbonyl oxygens of uL14 residues A133 and A136 (Fig. 6b, c, and Supplementary Fig. 8g, i, j) or the guanidinium moiety of eIF6 R61 (Fig. 6c and Supplementary Fig. 8f-h, j). Supporting the apparently weakened eIF6-uL14 interface, an influx of water molecules was observed after ~100–150 ns in three of the mutant simulation replicas, satisfying the H-bonding potential of the eIF6 S106 sidechain and uL14 A133 and A136 backbone nitrogens (Fig. 6b, d, and Supplementary Fig. 8k–o). These water molecules persisted at the interface throughout the remainder of the simulation, leading to the displacement of the eIF6 core relative to uL14, followed by partial solvation of their interaction interface (Fig. 6e, f, Supplementary Fig. 9). We conclude that comparative MD simulations of the WT and mutant complexes support the hypothesis that the SDS-related eIF6 N106S mutation disrupts the eIF6-uL14 interaction interface and ultimately leads to a local increase in its solvation, due to the lower propensity for the mutant to satisfy the H-bonding network with uL14. Over longer time scales this will likely lead to eIF6 disassembly from the 60S subunit.

### *EIF6* mutations rescue larval lethality of Sbds-deficient *Drosophila*

**Fig. 7:**
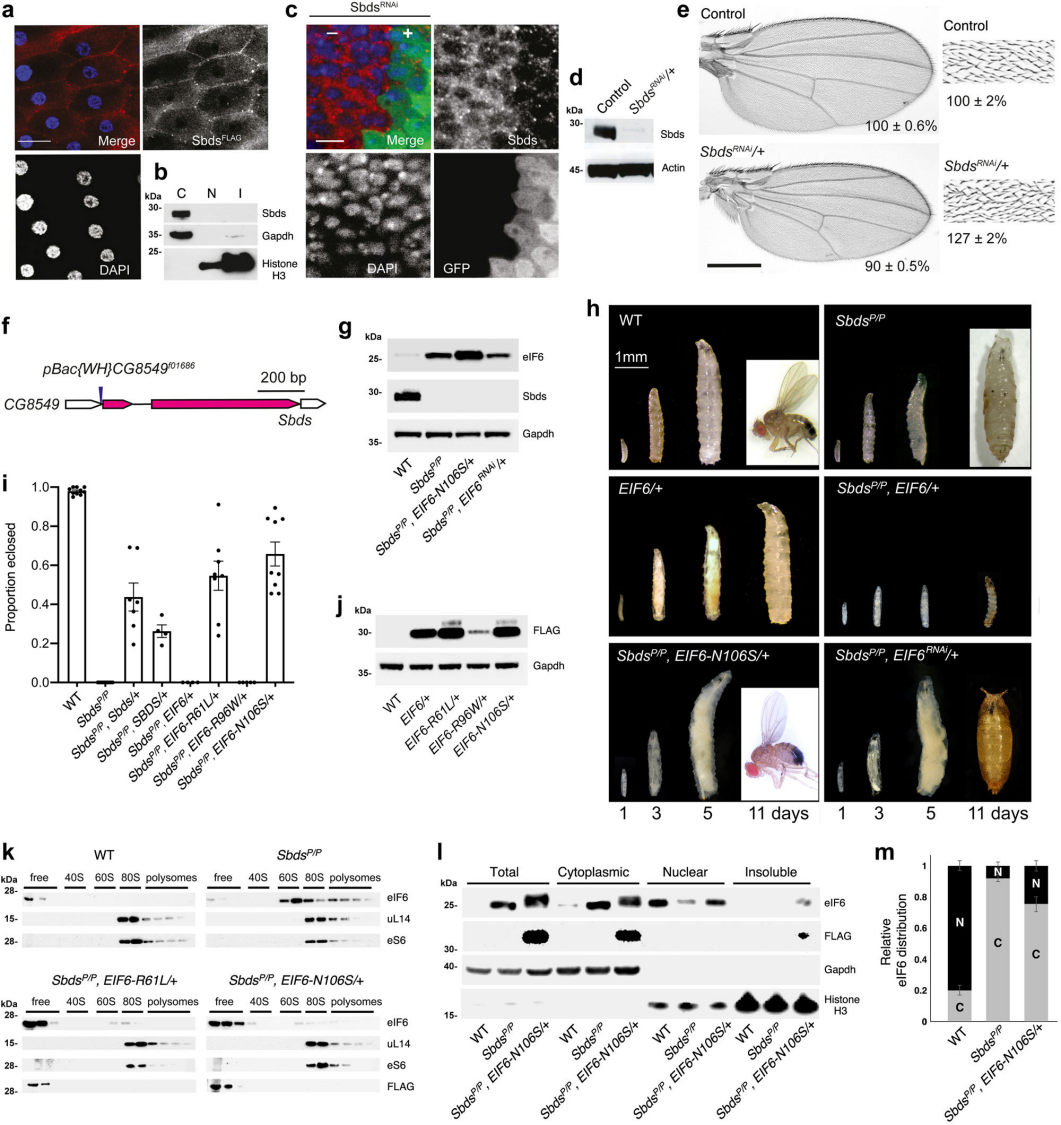
eIF6 missense mutations fully rescue the larval lethality of Sbds-deficient *D. melanogaster*. **a**–**c** Cytoplasmic localization of *Drosophila* Sbds by (**a**) immunostaining of FLAG-tagged Sbds (red) in ovarian follicle cells, the nucleus in blue (DAPI), scale bar: 10 μm, 3 replicates, *n* = 30; (**b**) immunoblotting of third instar *Drosophila* larval cytoplasmic (C), soluble nuclear (N) and insoluble nuclear (I) fractions (3 replicates); (**c**) indirect immunofluorescence of the third instar larval wing disc cells. Sbds (red) depleted by RNAi in posterior wing disc cells (marked with GFP); the nucleus is blue (DAPI), scale bar: 10 μm, 3 replicates, *n* = 30. **d** RNAi depletion of Sbds in third instar larval extracts revealed by immunoblotting (3 replicates). **e** *Sbds* is required for cellular growth. RNAi depletion of *Sbds* in developing wings versus control. Wing size (*n* = 15, *p-value* <0.0001, left) and bristle density (*n* = 10, *p-value* <0.0001, right) as a percentage (±s.e.) of control. Scale bar: 200 μm. A two-tailed student *t*-test was used. **f** *Drosophila Sbds* (CG8549) locus. PiggyBac-element insertion site (arrow) and *Sbds* coding region (magenta) are shown. **g** Indicated proteins revealed by immunoblotting of larval extracts from indicated genotypes (3 replicates). **h** eIF6-N106S mutation or eIF6 dose reduction rescues larval lethality of *Sbds*-deficient flies. Development at indicated time-points after egg laying is shown. Scale bar: 1 mm. **i** Genetic complementation of homozygous *Sbds*^*P/P*^ mutant flies (at least 4 replicates, minimum *n* = 156; error bars represent mean ± s.e). **j** SDS-related eIF6 mutant protein expression in WT larvae expressing eIF6 WT or missense mutants (3 replicates). **k** eIF6*-*N106S and R61L variants have lower affinity for the 60 S subunit. Larval extracts were fractionated by sucrose gradient sedimentation and proteins visualized by immunoblotting (3 replicates). **l** *EIF6-N106S* rescues the cytoplasmic redistribution of eIF6 in Sbds-deficient flies. Subcellular fractions of third instar larvae cells with the denoted genotypes were immunoblotted to visualize the indicated proteins (3 replicates). **m** Subcellular distribution of endogenous eIF6 in the denoted genotypes quantified by densitometry of (**l**). Error bars represent mean ± s.e.; 3 replicates. *Drosophila* strains and genotypes are listed in Supplementary Tables S2a, b.

We sought to test the general concept that somatic *EIF6* mutations can effectively rescue the deleterious effects of a hypomorphic germline *Sbds* mutation in a whole animal context by harnessing *Drosophila* genetics. We initially examined the subcellular localization and function of the *Drosophila* Sbds protein. *Drosophila* Sbds localized to the cytoplasm of ovarian follicle cells and in whole larvae (Fig. 7a, b) but did not colocalize with the mitotic spindle (Supplementary Fig. 10). In control experiments, Sbds protein expression was selectively lost in the posterior half of the wing disc in cells expressing *Sbds*^*RNAi*^ (marked with GFP) (Fig. 7c). We conclude that the *Drosophila* Sbds is a cytoplasmic protein, consistent with the localization of its mammalian and *Dictyostelium* counterparts^24,28^.

To examine the consequences of Sbds deficiency in *Drosophila*, we used RNAi to deplete Sbds in the imaginal disc of the developing wing (denoted *Sbds*^*RNAi/+*^ in Fig. 7d, e). Sbds depletion reduced the surface area of the adult wing by 10% compared with control (Fig. 7e). A corresponding 27% increase in cell number (as assessed by hair density) indicated a decrease in cell size (Fig. 7e). We next generated germline hypomorphic *Sbds* mutant (*Sbds*^*P/P*^) animals homozygous for the insertion of a PiggyBac-element transposon (*PBac{WH}CG8549*^*f01686*^) within the 5’ untranslated region of the *Sbds* (CG8549) gene, 18 nucleotides upstream of the start codon, on the third chromosome at cytological position 65C3 (Fig. 7f). In addition, we engineered homozygous *Sbds*^*P/P*^ mutants expressing six independent eIF6 missense variants, three (eIF6-C56R, eIF6-Y151H, and eIF6-V192F, all marked with an MYC tag) based on their strength as suppressors of the fitness defect of Sdo1-deleted yeast cells^25^ and their localization to the interface with uL14 (Supplementary Fig. 11a), together with three independent SDS-related mutants (eIF6-R61L, eIF6-R96W, and eIF6-N106S, all marked with a FLAG tag) (Fig. 4). Immunoblotting of cell extracts revealed a marked reduction in Sbds protein expression in homozygous *Sbds*^*P/P*^ mutants compared with WT (Fig. 7g). Phenotypically, compared with WT or *Sbds*^*P/P*^ mutants expressing eIF6-N106S-FLAG (Fig. 7h) or eIF6-C56R-MYC (Supplementary Fig. 11b), homozygous *Sbds*^*P/P*^ animals alone exhibited a severe growth defect, with only 5% of larvae surviving to the early pupal stage (Fig. 7h and Supplementary Fig. 11b, c). Remarkably, five of the *EIF6* missense mutant transgenes rescued the homozygous *Sbds*^*P/P*^ mutant to the adult stage (eIF6-C56R, 20.9%, *n* = 182; eIF6-R61L, 54.7%, *n* = 716, eIF6-N106S, 65.8%, *n* = 783, eIF6-Y151H, 71.7%, *n* = 350; eIF6-V192F, 38.2%, *n* = 164) (Fig. 7i and Supplementary Fig. 11b, d) while the eIF6-R96W mutant, that showed reduced expression compared with eIF6-R61L and eIF6-N106S (Fig. 7j), rescued to the pupal stage (Supplementary Fig. 11c). By contrast, overexpression of WT eIF6 induced lethality of WT animals at the third instar larval stage and further enhanced the larval lethality of *Sbds*^*P/P*^ animals at the early second instar larval stage (Fig. 7h, i). None of the *EIF6* missense mutant transgenes impaired the viability or fertility of WT *Drosophila* (Supplementary Fig. 11e). Furthermore, ~30% knockdown of *EIF6* expression by RNAi (Fig. 7g) significantly rescued the proportion of homozygous *Sbds*^*P/P*^ mutant animals that survived to the pupal stage (Fig. 7h and Supplementary Fig. 11c). Importantly, transgenic expression of *Drosophila* or human SBDS rescued the larval lethality of homozygous *Sbds*^*P/P*^ mutants to the adult stage (Fig. 7i), confirming that the mutant phenotype was indeed a consequence of Sbds deficiency and attesting to the conservation of SBDS protein function. Immunoblotting of sucrose gradient fractions revealed that expression of eIF6 missense mutants (eIF6-R61L, eIF6-N106S, and eIF6-C56R) rescued eIF6 retention on the 60S subunit (Fig. 7k and Supplementary 11f), the cytoplasmic retention of eIF6 (eIF6-N106S, eIF6-C56R) (Fig. 7l, m, and Supplementary Fig. 11g) and the functional impairment of ribosome assembly (Supplementary Figs. 11f and 12) observed in *Sbds*^*P/P*^ mutants compared with WT animals. However, the ~30% reduction of *EIF6* expression did not alter the proportion of free versus 60S-bound eIF6 protein (Supplementary Fig. 13). We conclude that reducing the dose of eIF6 or lowering the affinity of the interaction between eIF6 and the 60S subunit rescues the deleterious effects of a germline hypomorphic *Sbds* mutation in *Drosophila*. Taken together, these data are consistent with a conserved role for SBDS in catalyzing eIF6 release from cytoplasmic 60S ribosomal subunits in *Drosophila*.

## Discussion

In this study, we have identified acquired *EIF6* mutations as a common mechanism of somatic genetic rescue in SDS, a leukemia predisposition disorder caused by a germline defect in ribosome assembly that impairs the release of eIF6 from nascent 60S ribosomal subunits^20,23–25,28^. These somatic *EIF6* mutations rescue the primary molecular pathological defect in SDS in vivo, either by reducing the dose of eIF6 or by lowering the affinity of eIF6 for the 60S subunit.

The development of sensitive and reliable genetic tools has recently enabled the detection of mosaic somatic mutations and spontaneous chromosomal alterations in diverse tissues from normal individuals^10^. A growing number of studies have demonstrated that such somatic genetic modifications accumulate with age and participate in age-related disease, clonal expansion, and cancer development. However, in the context of Mendelian disease, de novo genetic events can counterbalance the deleterious effect of germline mutations, providing the somatically modified cells with a selective advantage compared with their non-modified counterparts. This phenomenon of SGR has been reported in Mendelian hematopoietic disorders where it promotes the clonal expansion of SGR positive cells detectable in blood^13^. In this study, ultra-deep targeted sequencing has revealed that genetic alterations in the *EIF6* gene that impact the stability or expression of eIF6 or its interaction with the 60S subunit represent a recurrent indirect mechanism of SGR in hematopoietic cells from SDS patients. In agreement with the reported accumulation of somatic genetic alterations over time in hematopoietic cells from normal individuals^5,6^, we found that the frequency of independent *EIF6* mutations in SDS positively correlates with increasing age. However, the frequency of somatic mutations over time in hematopoietic cells from normal individuals is still a matter of debate^10^. Strikingly, we detected *EIF6* mutant clones in 4 SDS patients below 10 years of age, one of whom was 3.4 years old. In addition, we detected multiple independent *EIF6* mutant clones (up to 8) in several SDS patients. Together these observations support the idea that the acquisition of somatic mutations in hematopoietic cells is more frequent than previously thought, as they have generally only been unveiled in a context where they provide a selective advantage and promote clonal expansion^10^.

*Sbds* deletion from mesenchymal stem cells in the mouse induces mitochondrial dysfunction, oxidative stress, and activation of the DNA damage response (DDR) in hematopoietic stem and progenitor cells (HSPCs)^41^. These data led to the proposal that mesenchymal inflammation promotes genotoxic stress in SDS HSPCs and drives the evolution of leukemia. However, the mutational signature in our analysis predominantly consists of C>T transitions (Fig. 2e) that characterize mutations that accumulate with age in normal individuals^5,6^, suggesting that the contribution of DDR pathways to the promotion of SGR in SDS bone marrow cells is limited (or virtually absent). Since somatic mutations also accumulate in tissues outwith the hematopoietic system^4,10^, it will be interesting to determine whether cellular clones with somatic *EIF6* mutations arise in other organs in SDS, a multi-system disorder caused by a germline ribosome assembly defect.

The hematological manifestations in SDS are highly heterogeneous in different individuals who carry identical germline *SBDS* mutations and may even fluctuate within a single individual over time^42^. However, we found no correlation between the presence and/or frequency of *EIF6* somatic mutations and the hematological parameters. Longitudinal analysis will be necessary to determine whether clonal expansion promoted by the acquisition of somatic *EIF6* mutations delays or abrogates the emergence of hematological complications such as aplastic anemia, myelodysplastic syndrome (MDS), or acute myeloid leukemia (AML). Clonal hematopoiesis and progression to poor prognosis MDS in SDS are associated with the acquisition of somatic *TP53* mutations^43,44^. Single-cell sequencing will be required to determine whether individual clones can carry both *EIF6* and *TP53* somatic mutations and whether these variants are mutually exclusive. Further studies are also warranted to examine the effects of *EIF6* and/or *TP53* mutant clones on disease outcome in SDS.

Recently Koh et al. reported an individual with clinical features of SDS in whom a de novo heterozygous missense *EIF6* mutation (c.182G>T; p.Arg61Leu (denoted R61L)) was identified by whole-exome sequencing of peripheral blood leukocytes and proposed to be disease-causing^39^. Intriguingly, the hematological abnormalities observed in this patient improved over time. Our analysis of fibroblasts from this individual (denoted SD-01) failed to identify a germline *EIF6* c.182G>T; R61L mutation. By contrast, we identified germline compound heterozygous mutations in the *SBDS* gene (c.183_184delTAinsCT; p.Lys62Ter and c.258+2T>C), associated with markedly reduced SBDS protein expression (Supplementary Fig. 14a) and an *SBDS* splicing anomaly (Supplementary Fig. 14b), consistent with the clinical diagnosis of SDS. We identified an increased ratio of 60S:80S subunits in extracts from SD-01 fibroblasts compared with control following sucrose sedimentation (Supplementary Fig. 14c, d) and reduced global protein translation as measured by OP-Puro incorporation (Supplementary Fig. 14e, f). Given our observation that somatic *EIF6* mutations are frequent in blood cells from SDS patients and can promote clonal expansion, these data suggested to us that rather than disease-causing, the *EIF6-R61L* mutation was an example of SGR counteracting the deleterious effect of a defect in ribosome assembly due to biallelic germline mutations in *SBDS*. Consistent with this hypothesis, the eIF6-R61L mutation rescued the fitness defect of Sdo1-deficient yeast cells (Fig. 5f and Supplementary Fig. 7), showed reduced cofractionation with the 60S subunit compared with wild type eIF6 (Fig. 7k and Supplementary Fig. 6), and fully rescued the larval lethality of Sbds-deficient *Drosophila* (Fig. 7i). We propose that the selective advantage provided by the somatic *EIF6-R61L* mutation promoted the expansion of the SBDS-deficient HSPCs to repopulate the hematopoietic system to a VAF close to 50% in peripheral blood DNA. Similar phenomena have been observed in other Mendelian hematopoietic disorders^14–16^.

**Fig. 8:**
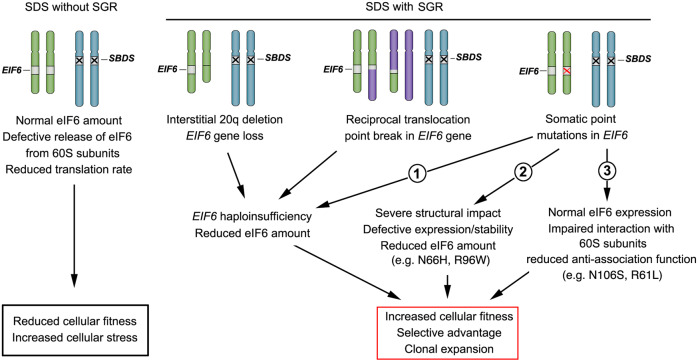
Schematic representation of EIF6 somatic genetic rescue (SGR) mechanisms in SDS. Chromosomal alterations (interstitial 20q deletion, reciprocal translocation), somatic nonsense or small indel mutations may cause *EIF6* hapoinsufficiency (denoted 1); other *EIF6* somatic point mutations may reduce eIF6 protein expression/stability (denoted 2) or impair the interaction of eIF6 with 60S subunits (denoted 3).

By combining ultra-deep *EIF6* sequencing, cytogenetic, structural, MD simulations, and functional analysis, our study provides evidence that distinct genetic *EIF6* alterations can rescue the germline ribosome assembly defect to promote clonal expansion in SDS hematopoietic cells and achieve SGR (Fig. 8). We confirmed the presence of an interstitial deletion in chromosome 20 that encompasses *EIF6* in hematopoietic cells from some individuals with SDS^29–31^. However, as the interstitial chromosomal deletion removed additional genes to *EIF6*, we were unable to formally conclude that expansion of del(20q) clones was a specific consequence of *EIF6* haploinsufficiency. The detection in hematopoietic cells from an SDS patient of a reciprocal translocation in which one of the breakpoints disrupted the *EIF6* gene while the other resided within a non-coding region strongly supports the idea that *EIF6* haploinsufficiency does indeed provide a selective advantage and promotes the clonal expansion of SBDS-deficient cells (Fig. 8). To our knowledge, SGR induced by a reciprocal translocation has not been previously reported^13^. Lastly, our ultra-deep sequencing analysis pinpointed the existence of frequent and distinct point mutations in the coding sequence of *EIF6* that promoted SGR. Interestingly, we detected several mutations that recurrently affected the same conserved residues. We distinguished three categories of *EIF6* point mutations: (1) nonsense and frameshift mutations that led to *EIF6* haploinsufficiency; (2) missense mutations affecting highly conserved amino-acids that strongly reduced eIF6 expression and/or stability and either impaired or indeed completely abrogated eIF6 function in vivo (Supplementary Fig. 7); (3) missense mutations that did not impair eIF6 expression but reduced its affinity for the 60S subunit (e.g. N106S, R61L, G14S) (Fig. 8). Our MD simulations, supported by in vivo functional analysis, demonstrate that the eIF6 N106S mutant provides a particularly potent selective advantage that is explained by the key structural role of residue N106 in mediating polar interactions between eIF6 and ribosomal protein uL14 on the intersubunit face of the 60S subunit.

In conclusion, our study demonstrates that spontaneous acquired mutations affecting the *EIF6* gene represent a frequent mechanism of indirect SGR of the germline defect in ribosome assembly in SDS. The demonstration that the recurrent missense mutation N106S promotes SGR by reducing the affinity of eIF6 for the 60S subunit provides a compelling in vivo rationale for the development of small molecules that mimic the effects of eIF6 suppressor mutations in reducing the affinity of eIF6 for the 60S subunit as disease-modifying therapeutics in SDS^25^. Lastly, our results support the notion that SGR might represent a universal phenomenon, more frequent than previously suspected, that influences the clinical evolution of diverse Mendelian disorders that are not restricted to the hematopoietic system. In addition, the phenomenon of SGR may also be frequent in non-inherited disorders and tissue regeneration as recently exemplified in chronic liver disease^45^. The continued improvement in sequencing technologies will likely permit the exploration of SGR in many other disorders in the near future.

While this paper was in revision, an independent study was published reporting clonal hematopoiesis due to acquired somatic *EIF6* mutations in patients with germline *SBDS* deficiency^46^.

## Methods

### Study approval

Informed and written consent was obtained from donors and patients. The study and protocols comply with the 1975 Declaration of Helsinki, as well as with the local legislation and ethical guidelines from the Comité de Protection des Personnes de l’Ile de France II and the French advisory committee on data processing in medical research. The INSERM Institutional Review Board also approved this study.

### Constructs with human *EIF6*

The coding sequence of WT or mutant human eIF6 was inserted in the linearized (BglII/NotI) p3X-FLAG-Myc-CMV-26 vector (Sigma) to express N-terminal FLAG-tagged eIF6 protein (Supplementary Table S3). The *EIF6* mutations were introduced by hemi-RT-PCR with specific primers (Supplementary Table S4). The PCR products and linearized p3X-FLAG-Myc-CMV-26 vector were assembled with NEBuilder® HiFi DNA assembly master mix (New England Biolabs). Nucleotide numbering reflects the cDNA sequence with +1 corresponding to the A of the ATG translation initiation codon in the reference sequence.

### Immunoblotting of human cell extracts

2 × 10^6^ HEK293T were transfected with 3 μg of vectors expressing FLAG-eIF6-WT or FLAG-eIF6-mutants by electroporation (Biorad) or lipofectamine 2000 (Invitrogen). Seventy-two hours post-transfection, cells were scraped, washed in PBS, and lysed for 20 min on ice in lysis buffer containing 50 mmol/L Tris (pH 8.0), 2 mmol/L EDTA, 1% Triton X100, 1% phosphatase inhibitor cocktails (Sigma), and protease inhibitor (Roche Applied Science, Indianapolis, IN) and centrifuged; the supernatant was harvested and protein concentration quantified using the Bradford assay. Whole-cell lysates were analyzed by immunoblotting with appropriate antibodies using the Odyssey® CLx Imaging System (LI-COR Biosciences) for quantification.

### Targeted *EIF6* sequencing by NGS (capture by hybridization approach) and genetic analysis

Genomic DNA was extracted from whole blood cells or bone marrow. Illumina compatible barcoded genomic DNA libraries were constructed according to the manufacturer’s sample preparation protocol (Ovation Ultralow V2, Nugen Technologies). Briefly, 400 ng to 3 µg of patient genomic DNA was mechanically fragmented to a median size of 200 bp using a Covaris. One hundred nanograms of double-strand fragmented DNA was end-repaired and adapters containing a Unique Dual Index barcode (IDT) were ligated to the repaired ends (one pair of barcodes per patient). Ligated DNA fragments were PCR amplified to obtain precapture barcoded libraries that are pooled at equimolar concentrations. The capture process was performed using the SureSelect reagents (Agilent), 750 ng of the pool of precapture libraries, and homemade biotinylated probes (as previously described in Benyelles et al.^47^ and Venot et al.^48^. The biotinylated single-stranded DNA probes were designed and prepared to cover a 123 kb chromosomal region including the *ElF6* gene on chromosome 20 (chr20:35,256,992-35,380,631, according to the GRCh38.p12 assembly of the human reference genome) or the *EIF6* cDNA was obtained by PCR amplification with primers located in the 3’ and 5’ UTR (Sequence (5’->3’) F: CGG GGC CTG AGG GAC GGA GG; R: ACA ACA GAG CAG GTT TTT GC). During the capture process, barcoded library molecules complementary to the biotinylated beads were retained by streptavidin-coated magnetic beads on a magnet and PCR amplified to generate a final pool of postcapture libraries covering the targeted genomic regions. Pools of these final libraries were prepared and sequenced either on an Illumina HiSeq2500 or NovaSeq6000 (Paired-End sequencing 130 + 130 on HiSeq, 100 + 100 bases on NovaSeq, production of ~60 million clusters per sample). After demultiplexing, sequences were aligned to the reference human genome hg19 using the Burrows-Wheeler Aligner^49^. The mean depth of coverage per sample was >=1000× to enable a more accurate Copy Number Variant Analysis. Downstream processing was carried out with the Genome Analysis Toolkit (GATK), SAMtools, and Picard, following documented best practices (http://www.broadinstitute.org/gatk/guide/topic?name=best-practices). Variant calls were made with the GATK Unified Genotyper. Variants at very low allele frequency were called by freebayes with the option -F 0,0005 (–min_alternate_fraction) (https://arxiv.org/abs/1207.3907). The annotation process is based on the latest release of the Ensembl database. Variants were annotated, analyzed, and prioritized using the Polyweb/PolyDiag software interface designed by the Bioinformatics platform of University Paris Descartes/Imagine Institute.

The sequence analysis dn/ds tool from UCSF (https://humangenetics.ucsf.edu/sequencing-tool/) was used to calculate dN/dS.

### Cytogenetics and CGH array

Agilent SurePrint G3 Cancer CGH+SNP 4x180K microarray (Agilent Technologies, Santa Clara, CA) was used for genomic copy number analyses according to manufacturers’ recommendations. Genomic positions are relative to the human genome Build NCBI37/hg19. Chromosomal preparation from bone marrow was performed using standard protocols and fluorescence in situ hybridization (FISH) was performed using Del (20q) Deletion Probe LPH 020 (Cytocell Ltd, Cambridge, UK) according to manufacturers’ recommendations.

### Determination of the translocation t(16;20)(q24;q11.2) breakpoints with chimeric reads

To accurately assess the breakpoint location of chromosome 20/chromosome 16 translocation, we extracted all the reads from chromosome 16 that contain a soft clip in the cigar and determined the position of the last aligned position. We then grouped all those putative break points according to their position to look for clustering. Finally we retained the candidate clusters where mates pointed to chromosome 20 only, and the *EIF6* region in particular, for visual inspection with IGV. The command used was: samtools view -q 1 sample.bam chr16 | cut -f3,4,6-8 | grep S | awk ‘{pos=$2; split($3,a,”[IMDSH]”); split($3,b,”[0-9]*“); nb=length(b); for (i=2; i<=nb; i++) if (b[i] ~ /[MD]/) pos=pos+a[i-1]; printf(“%s\t%s\t%s\t%s\t%s\t%s\n”,$1, pos-1, pos-1, $3, $4, $5)}’ | sort -k2,2n | bedtools merge -d 0 -c 5,6 -o distinct,distinct | grep -E ‘=,chr20|chr20,=‘ | grep -v -E ‘=,chr20,|chr20,=,’ | sort -k5,5n. Study of the reads assigned positions of the breakpoint to a position between 85,849,823 and 85,849,825 (HG19) on chromosome 16 and to a region ranging from 33,867,599 to 33,867,604 on chromosome 20. The translocation was supported by 10 reads on chromosome 16 in total. The boundary was supported by 6 reads where 3 were inter-chromosomal alignment. On chromosome 20, due to the read-depth greater than 1,800, the situation was less clear. However, we identified 10 inter-chromosomal alignment reads and 15 more reads supporting the breakpoint region. Similar analysis in 4 unrelated controls did not retrieve chimeric reads between chromosome 16 and 20.

### Sucrose gradient of human cell extracts

For ribosome fractionation, cytoplasmic extracts from HEK293T cells were prepared as already described^13^. For each sample 1 mg of extract was layered on a 10–50% sucrose gradient containing 20 mM Tris pH 7.6; 80 mM NaCl; 5 mM MgCl_2_; 1 mM DTT. The gradients were run in an SW41 Beckman rotor at 220,672 *g* for 140 min at 4 °C. Following centrifugation gradients were fractionated. Acquisition of the profiles was obtained using the UA6 UV/VIS detector from ISCO.

### Statistical analyses

Statistical analyses were performed on Prism (GraphPad Software) v9.1.2. Groups were analyzed by Student *t*-test as indicated and the difference was considered statistically significant for *p* < 0.05. Pearson correlation on Prism v9.1.2 (GraphPad Software) was used for correlation determination.

### *Dictyostelium* cell cultivation and transfection

Ax2 (DBS0235521) cells were grown in filter-sterilized HL5 (Formedium #HLE2) containing 200 µg/mL Dihydrostreptomycin (Sigma #D7253) in tissue culture dishes or in shaken suspension at 180 revolutions per minute at 22 °C. For transfection, cells were harvested from tissue culture plates and washed by centrifugation twice in ice-cold H40 buffer (40 mM HEPES, 1 mM MgCl_2_ pH 7.0). They were resuspended at 4 × 10^7^ cells/mL and 0.1 mL added to a pre-chilled electroporation cuvette (gap width 2 mm, Geneflow #E6-0062). 1–2 µg of supercoiled or restriction enzyme digested plasmid DNA was added and electroporated with two 350 V square wave pulses each of 8 ms duration delivered 1 s apart using a GenePulser Xcell (Bio-Rad)^27^. Ax2 cells expressing eIF6 or vector (pDM1203) alone were selected in 10 cm tissue culture dishes using 10 µg/mL G418 (Gibco Geneticin #10131-035). Clonal eIF6 knockout cell lines were selected in 96 well tissue culture plates (60 or 600 cells/well) in 0.15 mL of HL5 medium/well containing 10 µg/mL blasticidin (InvivoGen #ant-bl-1) and 10 µg/mL G418. After 7–12 days in the selection, confluent wells were harvested, the genomic DNA extracted (Quick-DNA™ Miniprep Kit, Zymo research #D3024) and screened by PCR using oligonucleotides DTO16 and DTO18 that bind to regions of the eIF6 genomic locus that are outside that of the knockout cassette (Supplementary Table S5)^28^.

### Plasmid construction

To make knockout vector pDT131 genomic DNA both proximal and distal to the *EIF6* gene were amplified by PCR using primer pairs DTO1/DTO9 and DTO2/DTO3 that introduced restriction enzyme sites for cloning (Supplementary Table S5). The PCR products were digested with ApaI or BamHI/SacII and cloned into pLPBLP on either side of the ‘floxable’ bsR cassette and the inserts verified by sequencing. *Dictyostelium* WT or mutant eIF6 expression plasmids were made by PCR amplification of the eIF6 coding sequence (DDB0234038) from Ax2 genomic DNA with the inclusion of BamHI and XbaI restriction sites. The digested PCR product was cloned into the corresponding restriction sites of the extrachromosomal vector pDM1203^50^. The eIF6 T56K, I58T, and N106S point mutations were introduced using PCR-mediated site-directed mutagenesis. Primer pairs Max15/Max16 were used for T56K, DTO28/DTO29 for I58T, and DTO30/DTO31 for N106S. All mutations were verified by sequencing.

### Cell lysis for ribosome profiles

Vegetative cells were treated with 100 µg/mL cycloheximide for 5 min prior to harvesting. Cells were pelleted by centrifugation and resuspended in buffer KK_2_ (16.5 mM KH_2_PO_4_, 3.9 mM K_2_HPO_4_, 2 mM MgSO_4_) plus 100 µg/mL cycloheximide. They were washed twice more in KK2, with a final wash in KK_2_ containing 100 µg/mL cycloheximide and 1× SigmaFast EDTA-free protease inhibitor cocktail (Sigma #S8830). The cell pellet was resuspended at 2 × 10^8^/mL in 50 mM HEPES pH 7.5, 40 mM Mg(CH_3_COO)_2_, 25 mM KCl, 5% sucrose, 0.4% IGEPAL® CA-630 (Sigma #I8896), 100 μg/mL cycloheximide, 1× SigmaFast EDTA-free protease inhibitor cocktail, 2 mM PMSF and lysed by passing through a 25 mm diameter Swin-Lok filter holder (GE Healthcare Life Sciences #420200) containing a prefilter (Millipore #AP1002500) together with a 5 µm nucleopore track-etched membrane (Whatman #110613). The lysate was cleared by centrifugation (8,000 *g* for 30 min at 4 °C) and the supernatant passed through a 33 mm Millex-® GV 0.22 µm PVDF filter unit (Millipore #SLGV033RS). The filtrate was divided into 1.4 mL aliquots after A_260_ determination, flash-frozen in liquid N_2_, and stored at −80 °C. All buffers were at 4 °C.

### Sucrose density gradients

Lysates were loaded onto a 10–40% (w/v) sucrose gradient in 50 mM Hepes pH 7.5, 25 mM K(CH_3_COO)_2_, 40 mM Mg(CH_3_COO)_2_ in Polyallomer 14 × 95 mm centrifuge tubes (Beckman). After centrifugation (Beckman SW40Ti rotor) at 260,900×*g* for 3 h at 4 °C, gradients were fractionated at 4 °C using a Gilson Minipuls 3 peristaltic pump with continuous monitoring (A_254_ nm) and polysome profiles recorded using a Gilson N2 data recorder. Proteins were precipitated from 0.5 mL fractions using 20% (v/v) trichloroacetic acid, separated on SDS-PAGE gels, and transferred to nitrocellulose membranes for immunoblotting.

### Subcellular fractionation

Vegetative cells in a mid-log phase were harvested, washed in KK2 buffer, and resuspended at 2 × 10^7^ cells/mL. One milliliter of cells was pelleted by centrifugation and lysed in NLB buffer (50 mM Tris-HCl pH 7.4, 5 mM Mg (CH_3_COO)_2_, 10% (w/v) sucrose, 2% (v/v) NP-40 by vortexing for 1 min. Nuclei were pelleted by centrifugation at 2300×*g* for 5 min at 4 °C and the supernatant was saved as the “crude cytoplasmic” fraction. The nuclear pellet, washed once in 1 mL of NLB and resuspended in 100 μL of NLB, was designated the “nuclear fraction.”

### Immunoblotting of *Dictyostelium* cell extracts

*Dictyostelium* cells were resuspended at 2 × 10^7^ cells/mL in 1× NuPAGE® sample buffer (Invitrogen #NP0007) containing 5% (v/v) 2-mercaptoethanol (Sigma #M6250) and heated at 95 °C for 3 min. 2 × 10^5^ cell equivalents were loaded per well of a NuPAGE™ 4–12% Bis-Tris gel and resolved in 1× MES SDS running buffer (Life technologies #NP0002). SeeBlue® Plus2 (Invitrogen #LC5925) or HiMark™ (ThermoFisher scientific #LC5699) prestained standards were used to calibrate each gel. The iBlot 2 Dry Blotting System (Invitrogen™ #IB21001) was used to transfer the proteins to nitrocellulose membranes (Invitrogen #IB23001). The membranes were blocked for 30 min in block buffer (PBS containing 0.1% (v/v) TWEEN®20 (Sigma #T2700) and 5% (w/v) dried skimmed milk powder). The primary antibody was diluted in block buffer and incubated with the blocked membrane for 2–4 h at room temperature or overnight at 4 °C. The membrane was washed for 10 min with gentle agitation in PBS-T buffer (PBS containing 0.1% (v/v) TWEEN®20) and this was repeated another 3 times with fresh PBS-T. The secondary antibody was diluted in block buffer and incubated with the washed membrane for 1–2 h at room temperature. The blot was developed in 1.5 mL of Immobilon® Western chemiluminescent HRP substrate (Millipore #WBKLS0500) according to the manufacturer’s instructions. The membranes were visualized with the ChemiDoc™ MP imaging system (Bio-Rad) using Image Lab software v6.0.1 (Bio-Rad).

### Yeast strains, plasmids, and primers

*S. cerevisiae* strains used in this study are listed in Supplementary Table S6, primers are listed in Supplementary Table S7, and plasmids in Supplementary Table S8. To create the *Sdo1*^*ts*^ strain, the conditional TS18 intein^28,40^ was amplified by PCR from plasmid pS5DH-G4MINT (gift from N. Perrimon) and inserted between the *SDO1* codons for K73 and C74 by homologous recombination. For the generation of Tif6-GFP mutants, site-directed mutagenesis of the pTIF6-GFP plasmid was performed using the Phusion High-Fidelity PCR kit (NEB) and transformed into XL1-Blue Electroporation-Competent cells (Agilent).

### Yeast growth assays

*sdo1*^*ts*^ yeast cells were grown in SD-URA liquid medium at 23 °C to stationary phase. 2 OD_600_ of cells were harvested and re-suspended in 500 µL mQ water. 2 µL of serial tenfold dilutions were spotted onto solid SD–URA medium and growth was assessed after 2 days of culture at 30 °C, or 3 days at 23 °C or 37 °C. Random sporulation analysis was performed as described previously^25^.

### Immunoblotting of yeast cell extracts

The *sdo1*^*ts*^ yeast cells were grown at 23 °C to an OD_600_ of 0.8–1 in SD–URA liquid medium. 1 OD_600_ of cells were harvested, washed, and re-suspended in 500 µL of mQ water. 50 µL of 1.85 M NaOH was added and the samples were incubated on ice for 10 min. Samples were further incubated on ice with 17.5 µL of 100% (w/v) of TCA and centrifuged for 5 min at 16,000×*g*. The pellet was washed with 500 µL of 80% acetone (v/v) and centrifuged for 5 min at 16,000×*g*. The supernatant was decanted and the resultant pellet air-dried. The pellet was resuspended in 1× NuPAGE LDS sample buffer (Thermo Fisher Scientific) containing 50 mM DTT prior to incubation at 70 °C for 10 min. Samples were separated using the NuPAGE 4–12% Bis-Tris gel (Thermo Fisher Scientific) containing 1× MES buffer (Thermo Fisher Scientific). Proteins were transferred from the gel to the nitrocellulose membrane using the iBlot 2 (Thermo Fisher Scientific) system. The nitrocellulose membrane was blocked with 5% (w/v) milk dissolved in PBST buffer (137 mM NaCl, 2.7 mM KCl, 4.3 mM Na_2_HPO_4_, 1.47 mM KH_2_PO_4_ with 0.1% (v/v) Tween 20) for 30 min. The blot was incubated with 1:1000 dilution of anti-eIF6 antibody (GenTex, #GTX117971) overnight at 4 °C followed by several 5 min washes with PBST buffer. The blot was incubated with 1:5000 dilution of anti-rabbit IgG HRP-linked antibody (Cell Signaling #7074) followed by several 5 min washes with PBST buffer. 1 mL of Luminol and 1 mL of Peroxide solution from the Western Chemiluminescent HRP Substrate kit (Immobilon) was incubated with the blot for 1 min. Proteins were visualized using the Bio-Rad Chemidoc MP imaging system.

### Yeast genetic complementation

These assays were performed as previously described^25^.

### Drosophila melanogaster strains and genetics

Flies were maintained using standard culture techniques. All crosses were performed at 25 °C unless otherwise stated. Fly strains and genotypes are described in Supplementary Table S2. *CG8549*^*f01686*^, *PBac{WH}CG8549[f01686]*, referred to here as *Sbds*^*P*^, is a homozygous lethal piggyBac transposase element insertion in the 5′ untranslated region of *CG8549*.

### Transgenic *Drosophila* lines

The coding sequences for WT *Drosophila Sbds* (NM_139800) and *EIF6* (NM_145105) were amplified by PCR from a *Drosophila* embryo cDNA library (gift from Simon Bullock) and cloned into pTWF (The Drosophila Gateway vector collection) to generate plasmids pUAS-Sbds-FLAG and pUAS-EIF6-FLAG. *EIF6* suppressor mutations, *EIF6C56R, EIF6Y151H*, and *EIF6V192F* were generated by PCR site-directed mutagenesis and sub-cloned into vector pPWM (The *Drosophila* Gateway vector collection) using the Gateway system (Invitrogen). Transgenic *pUAS-Sbds-FLAG, pUAS-EIF6-FLAG, pUAS-EIF6-C56R-MYC, p-UAS-EIF6-Y151H-MYC*, and *pUAS-EIF6-V192F-MYC* flies were generated by P element–mediated germline transformation^51^ into a *w*^*1118*^ strain by Genetic Services Inc. Three SDS-related *EIF6* mutations, *EIF6-R61L, EIF6-R96W*, and *EIF6-N106S* were generated by PCR site-directed mutagenesis and sub-cloned into vector pTWF and pPWM (*Drosophila* Gateway vector collection) using the Gateway system (Invitrogen). Transgenic *pUAS-EIF6-R61L-FLAG, pUAS-EIF6-R96W-FLAG, pUAS-EIF6-N106S-FLAG*, and *pUAS-EIF6-N106S-MYC* flies were generated by P element–mediated germline transformation into a *w*^*1118*^ strain by BestGene Inc. To generate flies expressing human SBDS, the coding sequence for human SBDS (NP_057122) was PCR amplified from a pRSETA-SBDS plasmid^24^ and sub-cloned into plasmid pTWF to generate plasmid pUAS-SBDS-FLAG. Transgenic *pUAS-SBDS-FLAG* flies were generated as described above. Primers are listed in Supplementary Table S9. Plasmids are listed in Supplementary Table S10.

### Antibodies

Antibodies are listed in Supplementary Table S11. Rabbit polyclonal antiserum was raised against *Drosophila* Sbds residues 1-252 and affinity-purified (Eurogentec).

### Protein expression and purification

Plasmid pSbds-His (encoding *Drosophila* Sbds, amino acids 1–252, fused at the C-terminus to 6x His residues) was transformed into *E. coli* C41(DE3) cells and Sbds-6xHis protein were purified by Ni-NTA affinity (GE Healthcare) and a Hiload 26/60 Superdex 75 column (GE Healthcare). Protein purity was assessed by SDS-PAGE and identity confirmed by mass spectrometry.

### Immunofluorescence

Wing discs dissected from third-instar larvae and ovaries dissected from adult female flies were fixed in 4% paraformaldehyde in PBS for 30 min at room temperature and processed for immunofluorescence (IF) staining as described^52,53^. For immunofluorescent staining of mitotic cells in neuroblasts, *Drosophila* brain squash slides were prepared as described^54^. Primary antibodies are listed in Supplementary Table S11. Alexa 488 (green)-conjugated or 563 (red)-conjugated or 647 (far red)-conjugated secondary antibodies (Invitrogen) were used at 1:1000 dilution. DNA was stained with DAPI in a mounting medium (Vector). Images were collected on a Zeiss LSM780 confocal system, imported to Image J v10.4 (Image J) and Photoshop CS5 (Adobe), and adjusted for brightness and contrast uniformly across entire fields.

### Immunoblotting of *Drosophila* cell extracts

*Drosophila* larval extracts were prepared by grinding ten third instar larvae in 150 μL NuPAGE LDS sample buffer (Invitrogen, #NP0007) using a pellet pestle (Eppendorf). Samples were cleared in a microfuge and denatured by heating at 95 °C for 10 min. Third instar larvae cells were fractionated using NE-PER nuclear and cytoplasmic extraction reagents (Thermo Scientific, #78833) according to the manufacturer’s instructions. Cell lysates were cleared in a microfuge and normalized for protein concentration using a BCA protein assay kit (Pierce, #23227). Samples were separated using SDS-PAGE for immunoblotting.

### Sucrose gradient sedimentation of *Drosophila* cell extracts

Ribosomal subunits were separated by sucrose density gradients as previously described^23^. Briefly, *Drosophila* third instar larvae were collected (typically 40 mg), washed with PBS, homogenized in lysis buffer A (20 mM HEPES pH 7.4, 50 mM KCl, 2.5 mM MgCl_2_, 0.5% (v/v) IGEPAL^®^ CA-630 (Sigma, #I8896), 0.5% (w/v) Sodium deoxycholate, 100 µg/mL cycloheximide (Sigma, #C7698) with complete EDTA-free protease inhibitors (Roche) and 0.5 U/mL RNase inhibitor (Invitrogen) and incubated for 15 min on ice. Lysates were cleared in a microcentrifuge. Equal amounts (typically 3–5 A_254_ U) were applied to a 10–40% (w/v) sucrose gradient in 14 mL of buffer B (20 mM HEPES at pH 7.4, 50 mM KCl, 2.5 mM MgCl_2_) and centrifuged (Beckman SW40 rotor) at 284,600×*g* for 2 h at 4 °C). Samples were loaded on a Brandel gradient fractionator, polysome profiles detected using an ÄKTAprime plus system (GE Healthcare), and 0.5 mL fractions were collected. Proteins were precipitated from sucrose gradient fractions with 10% (v/v) trichloroacetic acid (TCA), separated on SDS-PAGE gels, and transferred to PVDF membranes for immunoblotting.

### Measurement of protein synthesis

Protein synthesis in human fibroblasts was measured as described^23^. Briefly, OP-Puro (Invitrogen; final concentration 50 μM) was added to Cells growing at 70–80% confluence on a 12-well plate with culture medium (Dulbecco’s Modified Eagle Medium (DMEM, GibcoTM GlutaMAXTM), 10% fetal bovine serum (Sigma) and 1% Penicillin-Streptomycin (Pen-Strep, Sigma)) for 60 min. Cells were removed from wells and washed twice with ice-cold Ca^2+^ and Mg^2+^ free phosphate-buffered saline (PBS) (Invitrogen) with 100 μg/ml cycloheximide. Cells were fixed and permeabilized using the Cytofix/Cytoperm Fixation Permeabilization Kit (BD Biosciences). Azide-alkyne cycloaddition was performed using the Click-iT Cell Reaction Buffer Kit (Invitrogen) with azide conjugated to Alexa Fluor 488) at 5 μM final concentration. Following the Click-iT reaction, cells were washed twice in PBS supplemented with 2% fetal bovine serum, resuspended in PBS, and analyzed by flow cytometry (Becton Dickinson LSR Fortessa analyzer). Flow cytometry data analysis was performed using FlowJo v10.7 (FlowJo, Ashland, OR). Relative rates of protein synthesis were calculated by normalizing OP-Puro signals to control cells after subtracting background fluorescence (cells without OP-Puro).

### cDNA sequencing

For RT-PCR of human *EIF6* and *SBDS*, total RNA from patient and control primary fibroblasts was extracted using RNeasy Mini Kit (Qiagen, #74104) according to the manufacturer’s instructions. Reverse transcription was performed using SuperScript™ II Reverse Transcriptase (Invitrogen, #18064), and cDNAs were used as templates to amplify the full sequence of the *EIF6* and *SBDS* genes. Primers used for PCR are listed in Supplementary Table S4. PCR products were gel purified and cloned into pCR™-Blunt II-TOPO® (Invitrogen, # 45-0245) for sequencing.

## Molecular dynamics simulations

### System setup

The atomic model for MD simulations was based on the cryo-EM structure of the human 60S-eIF6 complex at 2.4 Å resolution (PDBID: 7OW7). The protein-RNA complex comprised: (i) eIF6 residues M1-N225; (ii) eL24 residues M1-K60; (iii) uL3 residues A45-P82, P206-T223 and H275-R378; (iv) uL14 residues S10-A140; and (v) 28S rRNA bases A4589-G4639, G4660-U4677, and A4473-U4482. The system setup was carried out using the CHARMM-GUI web server^55–57^. Proteins and RNA were inserted into a cubic box (dimension 11.2 nm), allowing a minimum of 1 nm distance from the box edges. Solvation was performed using TIP3P water. Sufficient potassium ions were added to neutralize the excess system charge, and potassium and chloride ion pairs were added to achieve a physiologically representative salt concentration in the system of 0.1 M.

### Simulation protocol

All simulations were performed using GROMACS v2019.6^58^ with the CHARMM36 additive force field^59^ algorithm. Energy minimization was performed using the steepest descent algorithm (<5000 steps) to remove steric clashes, and a 4 ns equilibration phase followed with all protein and RNA atoms were position-restrained with gradually reducing force constants to relax the system, ranging from 400 to 40 kJ mol^−1^nm^−2^. All dihedral angles were restrained during equilibration using a force constant of 4 kJ mol^−1^ nm^−2^. Production simulations were carried out in the NPT ensemble for 500 ns in triplicate for all systems. During production runs, position restraints were applied to uL3 (backbone atoms of residues P82, P206, T223, and H275) and the 28S RNA (main chain atoms of the 5’ and 3’ terminal bases A4589, G4639, G4660, U4677, and A4473-U4482) to maintain the tertiary structure of uL3 and prevent unfolding of the 28S rRNA. We also ran an additional control set of simulations (4 replicas) of the mutant with the 28S rRNA fully fixed (Fc = 1000 kJ mol^−1^nm^−2^), which produced similar results. A 2 fs integration time step was used and trajectory frames were written every 20 ps. All covalent bond hydrogens were constrained using the LINCS algorithm^60^. Long-range electrostatics were treated with the Particle-Mesh-Ewald algorithm using a real-space cutoff of 1.2 nm^61^. Lennard-Jones interactions were smoothly switched off between 1.0 and 1.2 nm. The Nosé-Hoover thermostat was utilized to maintain the temperature at 303.15 K with a coupling constant of 1 ps^62,63^. Protein and RNA were coupled separately from the solvent. Isotropic pressure coupling was applied at 1 bar using the Parrinello-Rahman barostat with a coupling constant of 5 ps and compressibility of 4.5 × 10^−5^ bar^−1^ ^63,64^.

### Simulation analysis

The VMD v1.9.4 software was used for trajectory visualization and figure preparation^65^. All analysis was performed using integrated tools within the GROMACS package v2019.6^58^. The Grace plotting tool v.5.1.25 and the GNU Image Manipulation Program (GIMP) v2.10.24 were utilized to visualize the plots.

### Reporting summary

Further information on research design is available in the Nature Research Reporting Summary linked to this article.

## Supplementary information

Supplementary Information

Dataset 1. List of SDS patients

Dataset 2. List of all coding *EIF6* variants CADD scores

Dataset 3. BAFs values of *EIF6* SNPs

Description of additional supplementary files

Reporting Summary

## Data Availability

The cryo-EM density map has been deposited in the Electron Microscopy Data Bank under accession code EMD-13094. The corresponding atomic coordinates have been deposited in the Protein Data Bank under accession code 7OW7. Sequencing data is not publicly available due to the possibility of compromising privacy. The sequence data are available under restricted access for ethical reasons, access can be obtained by request by contacting P. Revy (patrick.revy@inserm.fr). The timeframe for response to requests is of 2 weeks, with no restriction to data use. Source data are provided with this paper.

## Source data

Source Data File
