# Publisher Correction: Somatic genetic rescue of a germline ribosome assembly defect

**DOI:** 10.1038/s41467-022-31316-1

**Published:** 2022-06-22

**Authors:** Shengjiang Tan, Laëtitia Kermasson, Christine Hilcenko, Vasileios Kargas, David Traynor, Ahmed Z. Boukerrou, Norberto Escudero-Urquijo, Alexandre Faille, Alexis Bertrand, Maxim Rossmann, Beatriz Goyenechea, Li Jin, Jonathan Moreil, Olivier Alibeu, Blandine Beaupain, Christine Bôle-Feysot, Stefano Fumagalli, Sophie Kaltenbach, Jean-Alain Martignoles, Cécile Masson, Patrick Nitschké, Mélanie Parisot, Aurore Pouliet, Isabelle Radford-Weiss, Frédéric Tores, Jean-Pierre de Villartay, Mohammed Zarhrate, Ai Ling Koh, Kong Boo Phua, Bruno Reversade, Peter J. Bond, Christine Bellanné-Chantelot, Isabelle Callebaut, François Delhommeau, Jean Donadieu, Alan J. Warren, Patrick Revy

**Affiliations:** 1 Cambridge Institute for Medical Research, Cambridge Biomedical Campus Keith Peters Building, Hills Rd, Cambridge, United Kingdom; 2 Wellcome Trust-Medical Research Council Stem Cell Institute, Jeffrey Cheah Biomedical Centre, Puddicombe Way, Cambridge Biomedical Campus, Cambridge, UK; 3 Department of Haematology, University of Cambridge School of Clinical Medicine, Jeffrey Cheah Biomedical Centre, Puddicombe Way, Cambridge Biomedical Campus, Cambridge, UK; 4 Université de Paris, Imagine Institute, Laboratory of Genome Dynamics in the Immune System, Equipe Labellisée Ligue contre le Cancer, INSERM UMR 1163, Paris, France; 5 INSERM Unité Mixte de Recherche 1163, Structure Fédérative de Recherche Necker INSERM US24/CNRS UMS3633, Genomic Core Facility, Paris Descartes-Sorbonne Paris Cité University, Imagine Institute, Paris, France; 6 French Neutropenia Registry, Assistance Publique-Hôpitaux de Paris, Trousseau Hospital, Paris, France; 7 Institut Necker Enfants Malades, Paris, France; 8 INSERM, U1151, Université Paris Descartes Sorbonne Cité, Paris, France; 9 Université Paris Descartes, Faculté de Médecine Sorbonne Paris Cité, Paris, France; 10 Service de cytogénétique, Hôpital Necker, Assistance Publique-Hôpitaux de Paris, Paris, France; 11 Sorbonne Université, Inserm, Centre de Recherche Saint-Antoine, AP-HP, Hôpital Saint-Antoine, Hématologie Biologique, Paris, France; 12 INSERM Unité Mixte de Recherche 1163, Bioinformatics Platform, Paris Descartes-Sorbonne Paris Cité University, Imagine Institute, Paris, France; 13 Department of Paediatrics, KK Women’s and Children’s Hospital, Singapore, Singapore; 14 SingHealth Duke-NUS Genomic Medicine Centre, Singapore, Singapore; 15 Genome Institute of Singapore, A*STAR, Biopolis, Singapore, Singapore; 16 Bioinformatics Institute (A*STAR), Singapore, Singapore; 17 Department of Biological Sciences, National University of Singapore, Singapore, Singapore; 18 Department of Genetics, Pitié-Salpêtrière Hospital, Sorbonne University, Paris, France; 19 Sorbonne Université, Muséum National d’Histoire Naturelle, UMR CNRS 7590, Institut de Minéralogie, de Physique des Matériaux et de Cosmochimie, IMPMC, Paris, France; 20 Service d’Hémato-Oncologie Pédiatrique, Assistance Publique-Hôpitaux de Paris Hôpital Trousseau, Registre des neutropénies-Centre de référence des neutropénies chroniques, Paris, France; 21 Present Address: PolyProx Therapeutics, Babraham Research Campus, Cambridge, UK; 22 Present Address: MRC Laboratory of Molecular Biology, Francis Crick Avenue, Cambridge Biomedical Campus, Cambridge, UK

**Keywords:** Mutation, Next-generation sequencing, Ribosome, Haematological diseases

Correction to: *Nature Communications* 10.1038/s41467-021-24999-5, published online 19 August 2021.

The original version of this article contained errors in equal contribution attributions that were inadvertently introduced during typesetting. These errors have now been corrected in both the PDF and HTML versions of the article.

