# Supplementary material for: Somatic genetic rescue of a germline ribosome assembly defect
Source: Nat Commun. 2021 Aug 19;12:5044. doi: 10.1038/s41467-021-24999-5 (PMC8377010; doi:10.1038/s41467-021-24999-5)
Supplement: Supplementary file 1 — Supplementary Information [file 41467_2021_24999_MOESM1_ESM.pdf]

Somatic genetic rescue of a germline ribosome assembly defect

List of Supplementary data

Supplementary Figure 1: FISH probes.
Supplementary Figure 2: Cumulative VAF vs age and cumulative VAF vs mutation count.
Supplementary Figure 3: Correlation between *EIF6* mutation and blood parameters.
Supplementary Figure 4: Sequence alignment of eIF6.
Supplementary Figure 5: FACS analysis of *EIF6* transfection in HEK293T cells.
Supplementary Figure 6: Reduced cofractionation of the Tif6-R61L missense variant with the 60S subunit in yeast.
Supplementary Figure 7: Functional characterization of SDS-related *TIF6* mutant alleles.
Supplementary Figure 8: Contacts at the uL14-eIF6 interaction interface
Supplementary Figure 9: Analysis of the stability of the uL14-eIF6 interaction interface.
Supplementary Figure 10: Cytoplasmic localization of Sbds in *Drosophila* mitotic cells.
Supplementary Figure 11: *EIF6* missense mutations that map to the interface with the 60S ribosomal subunit rescue germline *Sbds* deficiency in *Drosophila*.
Supplementary Figure 12: *EIF6-R61L* and *N106S* rescue the ribosome assembly defect in Sbds-deficient flies.
Supplementary Figure 13: *EIF6* depletion does not increase the amount of free eIF6 in Sbds-deficient *Drosophila* cells.
Supplementary Figure 14: Impaired translation due to SBDS protein deficiency in SD-01 patient fibroblasts.
Supplementary Data 1: List of SDS patients (Excel file).
Supplementary Data 2: CADD scores of all *EIF6* SNVs (Excel file).
Supplementary Data 3: SNPs /BAF (Excel file).
Supplementary Table S1: Frequency of eIF6 mutants in gnomAD, COSMIC and TCGA.
Supplementary Tables S2a, S2b: *Drosophila* genotypes and strains.
Supplementary Table S3: Plasmids (Human).
Supplementary Table S4: Oligonucleotides (Human).
Supplementary Table S5: Oligonucleotides (*Dictyostelium*).
Supplementary Table S6: Strains (yeast).
Supplementary Table S7: Oligonucleotides (yeast).
Supplementary Table S8: Plasmids (yeast).
Supplementary Table S9: Oligonucleotides (*Drosophila*).
Supplementary Table S10: Plasmids (*Drosophila*).
Supplementary Table S11: Antibodies.

Supplementary Figure 1

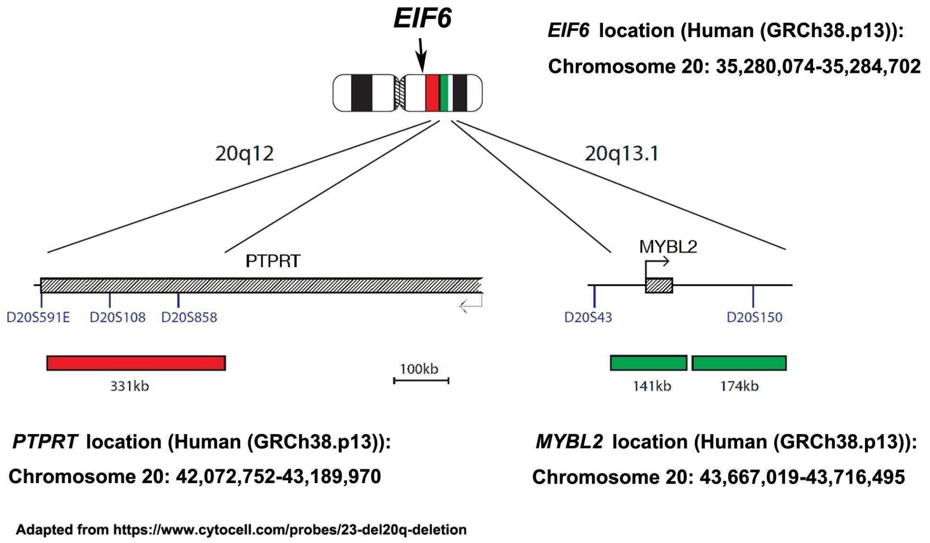

Suppl. Fig.1. FISH probes. Schematic representation of the FISH probes used to detect interstitial deletion on the q-arm of chromosome 20 and physical locations of the genes *PTRPT* and *MYBL2* (detected by FISH probes) and *EIF6*.

Supplementary Figure 2

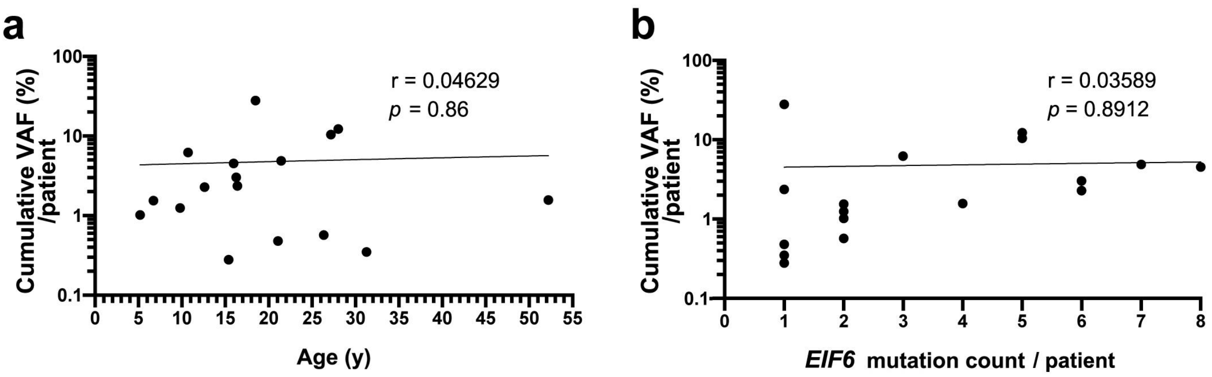

Suppl. Fig. 2. Cumulative VAF vs age and cumulative VAF vs mutation count. No correlation between cumulative *EIF6* VAF and age (**a**) or *EIF6* mutation count (**b**) in SDS. P-values and Pearson correlations are indicated.

Supplementary Figure 3

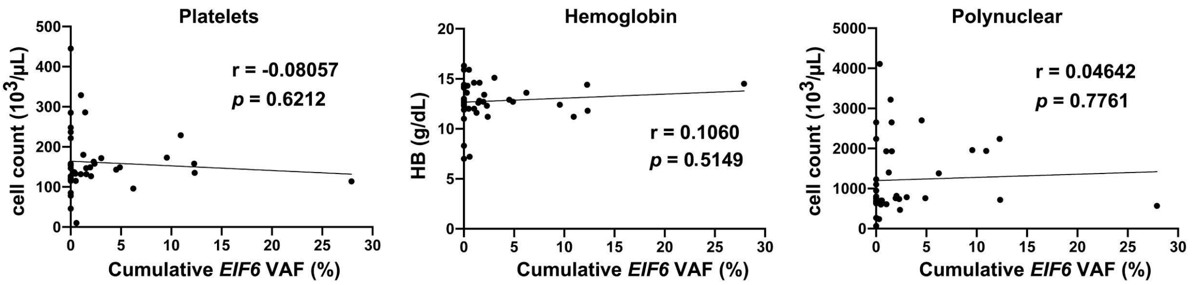

Suppl. Fig. 3. **Correlation between *EIF6* mutation and blood parameters** Platelets, hemoglobin and white cell counts in patients as a function of cumulative *EIF6* VAF. P-values and Pearson correlations are indicated.

Supplementary Figure 4

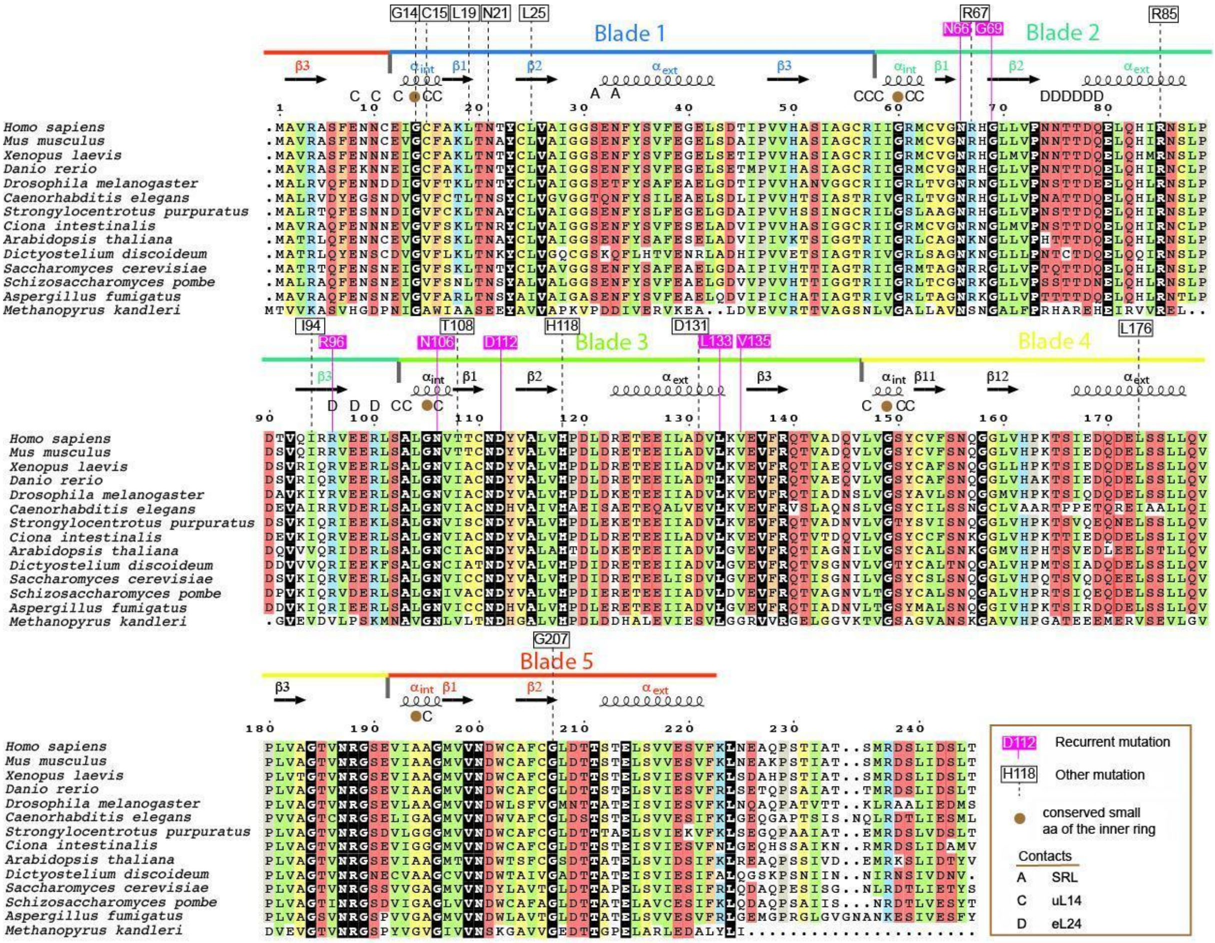

Suppl. Fig. 4. Sequence alignment of eIF6. Residues recurrently mutated in SDS are highlighted in pink. Other amino-acids mutated in SDS are highlighted in black boxes. Figure was drawn using ESPript[1].

Supplementary Figure 5

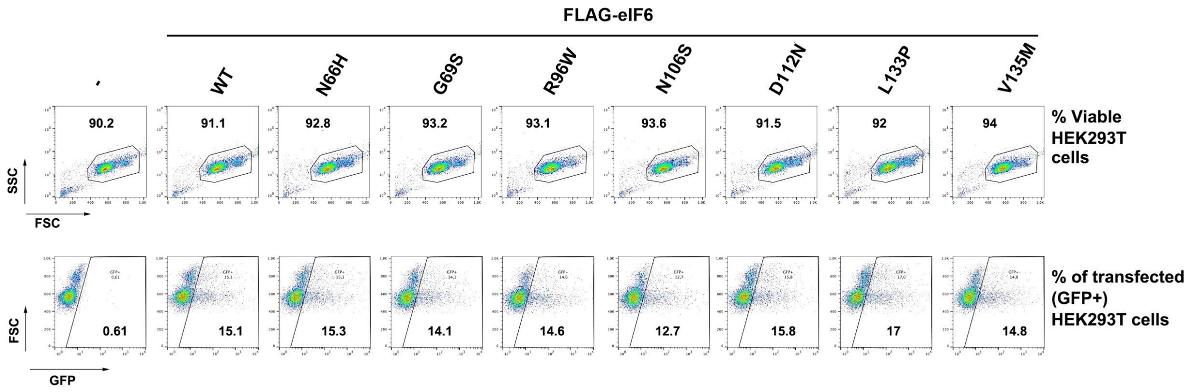

Suppl. Fig. 5. FACS analysis of *EIF6* transfection in HEK293T cells. FACS analysis of cell viability (gated on SSC/FCS; up) and efficiency of transfection (GFP+; down) in viable HEK293T cells transfected with the different eIF6-expressing constructs. Cell viability as well as the percentage of transfected cells were similar in all conditions.

Supplementary Figure 6

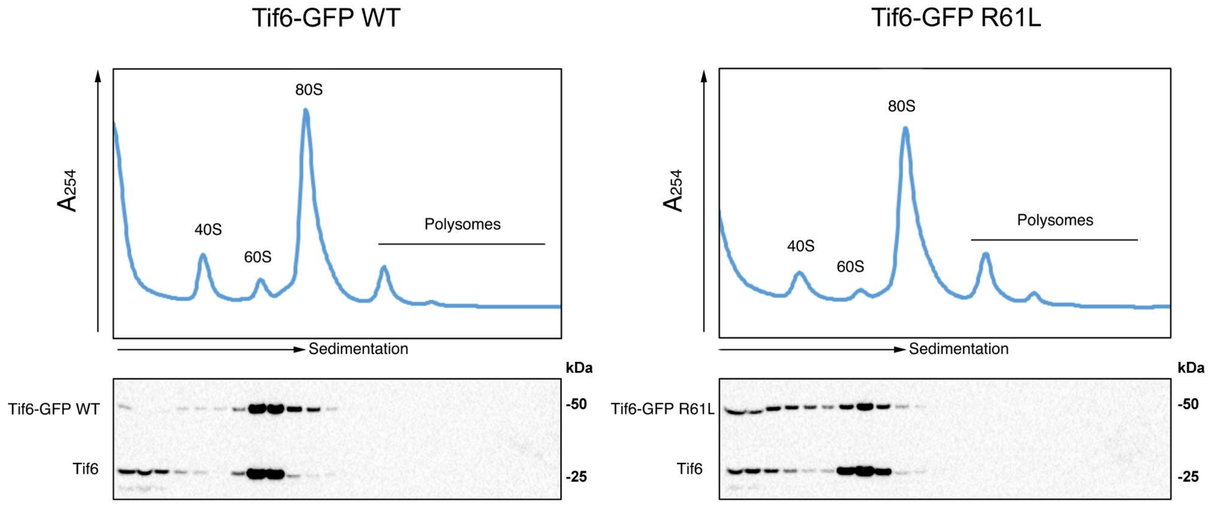

Suppl. Fig. 6. Reduced cofractionation of the Tif6-R61L missense variant with the 60S subunit in yeast. Extracts from *Sdo1*^{ts} cells (grown at 23 °C) expressing Tif6-GFP WT or Tif6-GFP R61L were fractionated by sucrose gradient sedimentation and Tif6 protein visualized by immunoblotting (2 replicates).

Supplementary Figure 7

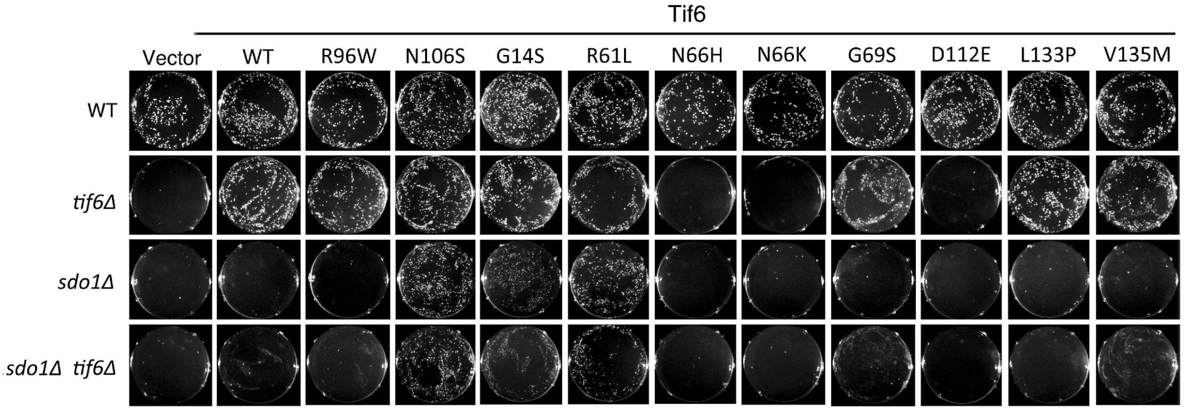

Suppl. Fig. 7. Functional characterization of SDS-related *TIF6* mutant alleles. Diploid *SDO1 sdo1Δ TIF6 tif6Δ* cells were transformed with empty vector, WT Tif6 or the indicated Tif6 mutants, sporulated and plated onto appropriate selective media for 3 days at 30 °C (see **Methods**) (2 replicates).

Supplementary Figure 8

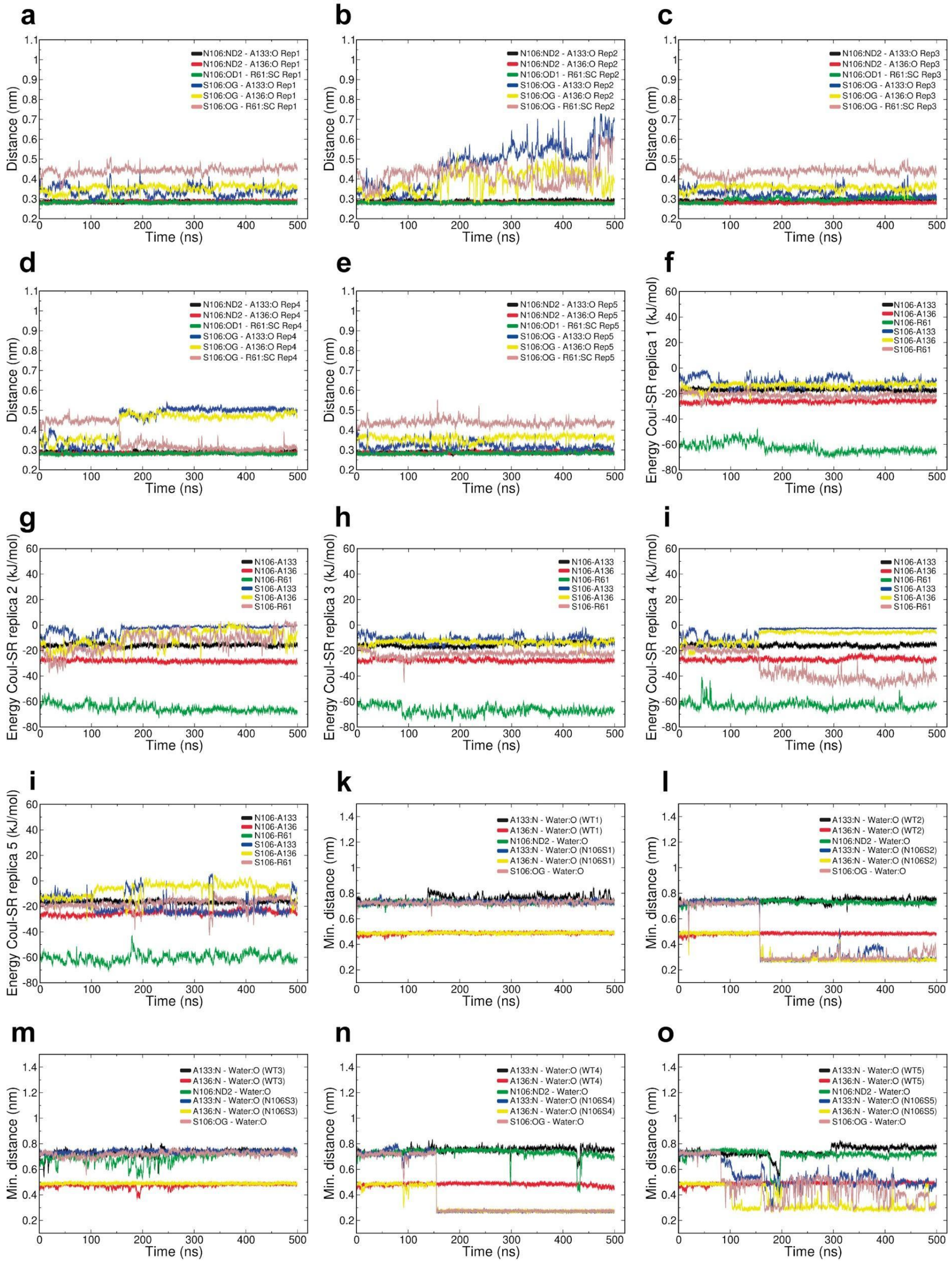

Suppl. Fig. 8. Contacts at the uL14-eIF6 interaction interface. a-e Distances (nm) and electrostatic interactions (**f-j)** between the indicated atoms of eIF6 WT and mutant (residues N106, S106 and R61), and uL14 (residues A133, A136) or water (**k-o**).

Supplementary Figure 9

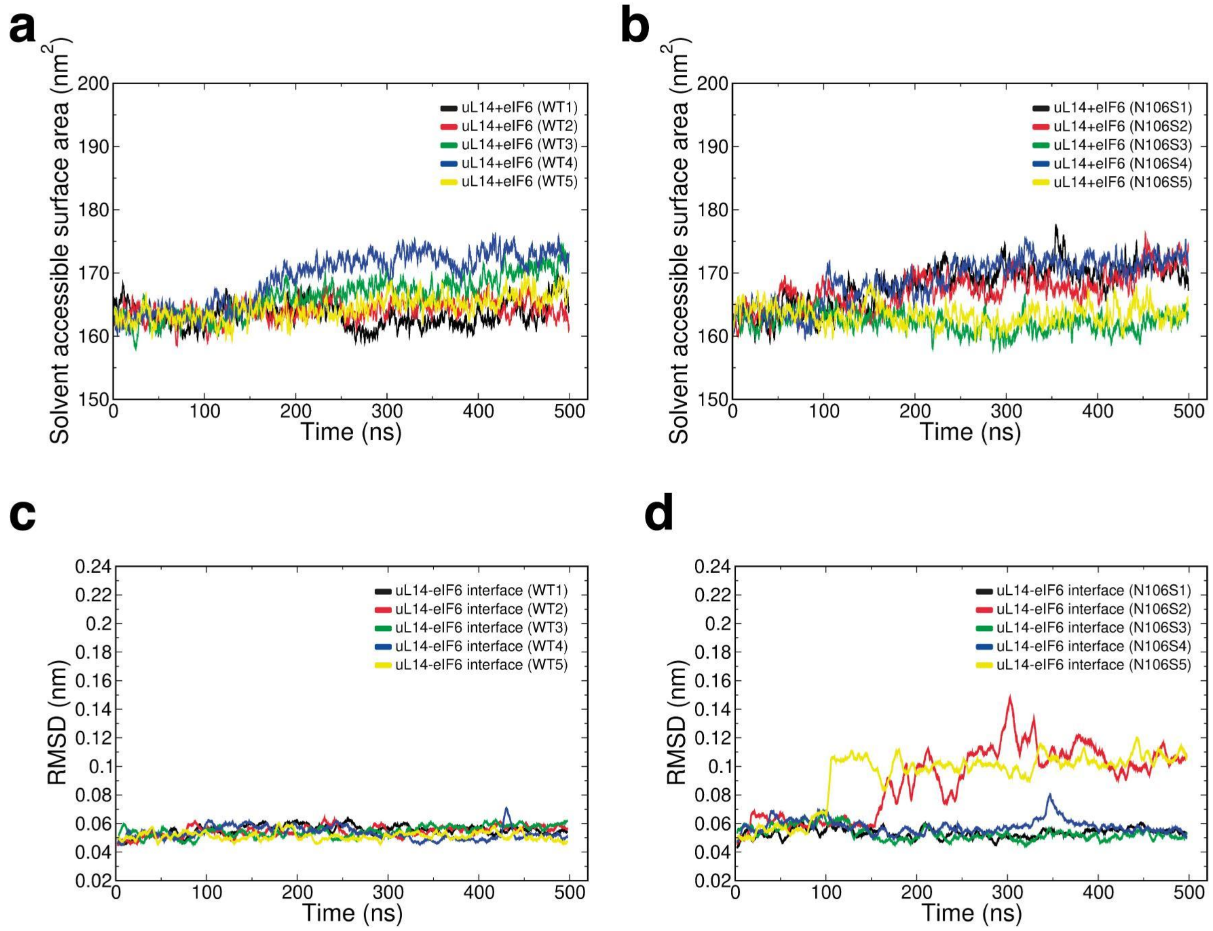

Suppl. Fig. 9. Analysis of the stability of the uL14-eIF6 interaction interface. a Solvent accessible surface area of uL14-eIF6 complex of the WT and mutant **(b)** simulations. RMSD of the backbone atoms of the uL14-eIF6 interface in the WT (**c**) and mutant (**d**).

Supplementary Figure 10

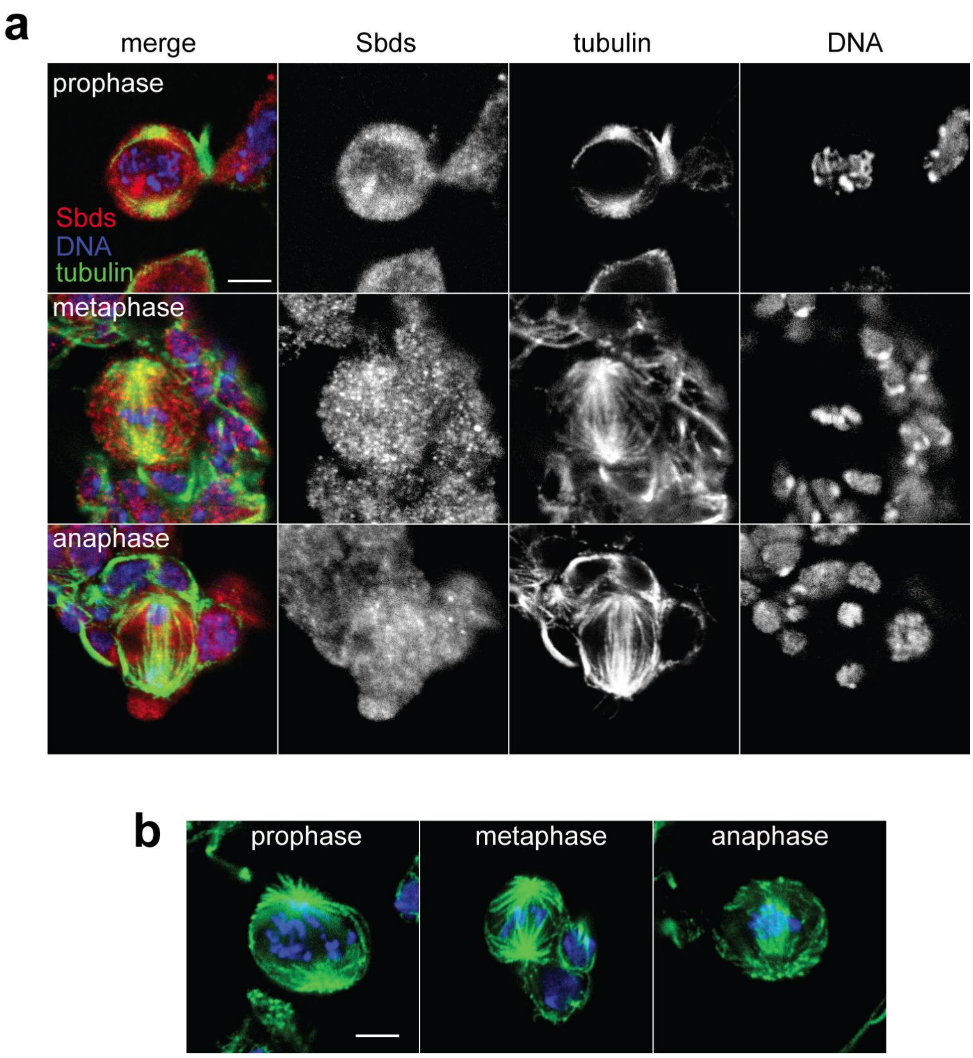

Suppl. Fig. 10. Cytoplasmic localization of Sbds in *Drosophila* mitotic cells. a Neuroblasts from WT *Drosophila* larvae showing no colocalization of Sbds (red) with microtubules (green, visualized with anti α-tubulin antibody) in mitotic cells (3 replicates).. **b** Neuroblasts from *Sbds*$^{P/P}$ mutant larvae show no defects in spindle stabilization in mitotic cells (3 replicates). Microtubules are in green, DNA in blue. Scale bars, 10 µm.

Supplementary Figure 11

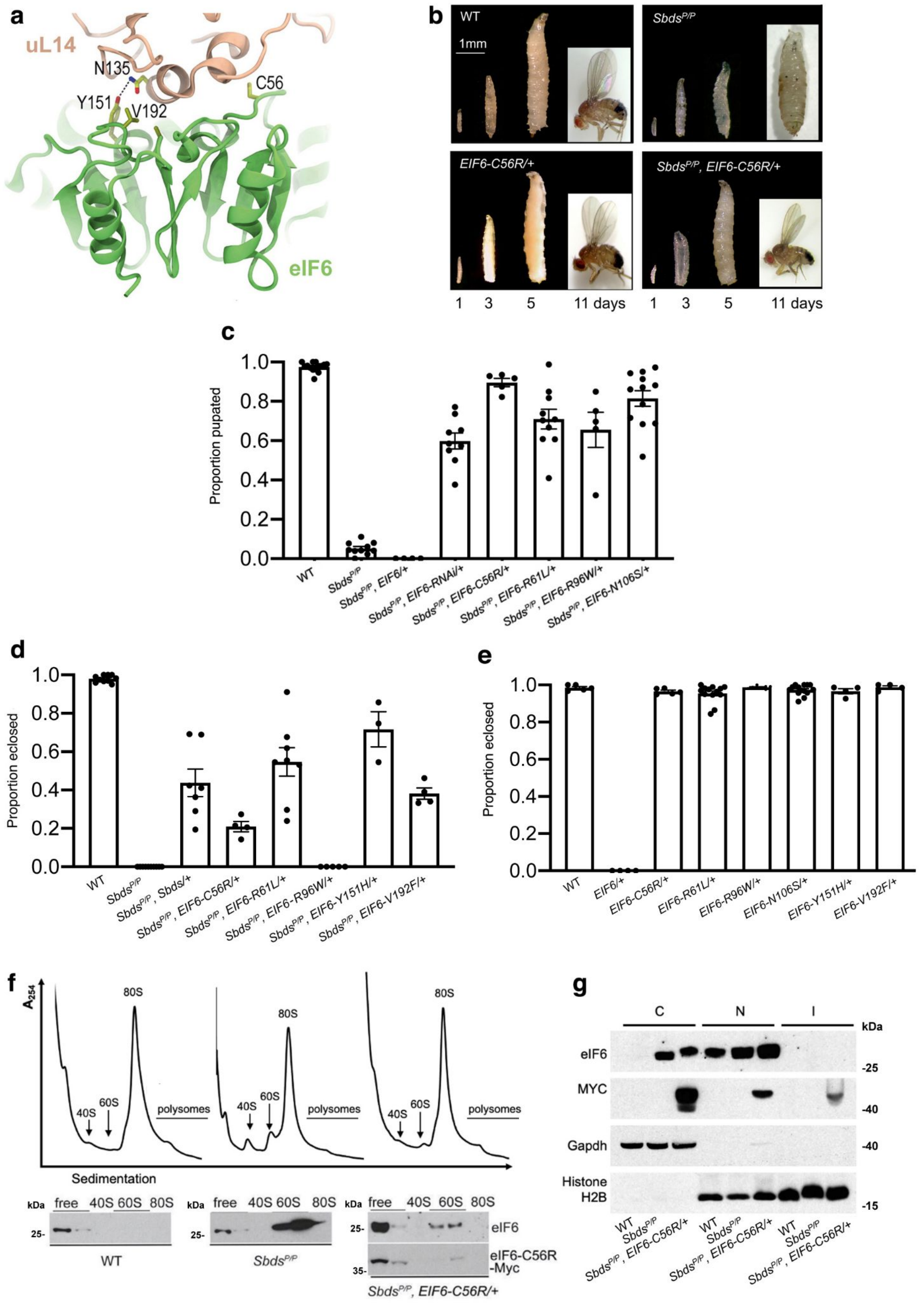

Suppl. Fig. 11. *EIF6* missense mutations that map to the interface with the 60S ribosomal subunit rescue germline *Sbds* deficiency in *Drosophila*. a eIF6 residues C56, V192 and Y151 lie at the interface with uL14. eIF6 is colored green; uL14, salmon. (PDBID: 70W7). **b-d** Knockdown of eIF6 expression by RNAi or overexpression of *EIF6* missense mutations rescues the larval lethality of *Sbds* deficient *Drosophila*. Development of the indicated fly genotypes at the specified time points after egg laying (3 replicates) (**b**) and the proportion that pupated (error bars indicate s.e.; at least 4 replicates with minimum n = 156 (**c**) or eclosed (at least 3 replicates with minimum n = 164) (**d**). **e** Expression of *EIF6* WT but not *EIF6* missense variants induces lethality in WT *Drosophila*. The

proportion of the indicated genotypes that eclosed is shown (error bars indicate s.e.; at least 4 replicates with minimum *n* = 150). **f** *EIF6-C56R* rescues the ribosome assembly defect in Sbds-deficient flies. Polysome profiles from the indicated fly genotypes (3 replicates). The indicated proteins were visualized by immunoblotting (3 replicates). **g** *EIF6-C56R* rescues the cytoplasmic redistribution of eIF6 in Sbds-deficient flies. Subcellular fractions of third instar larval cells with the indicated genotypes were immunoblotted to visualize the indicated proteins. C, cytoplasmic, N, nuclear; I, insoluble nuclear fraction containing nucleoli and chromatin (3 replicates).

Supplementary Figure 12

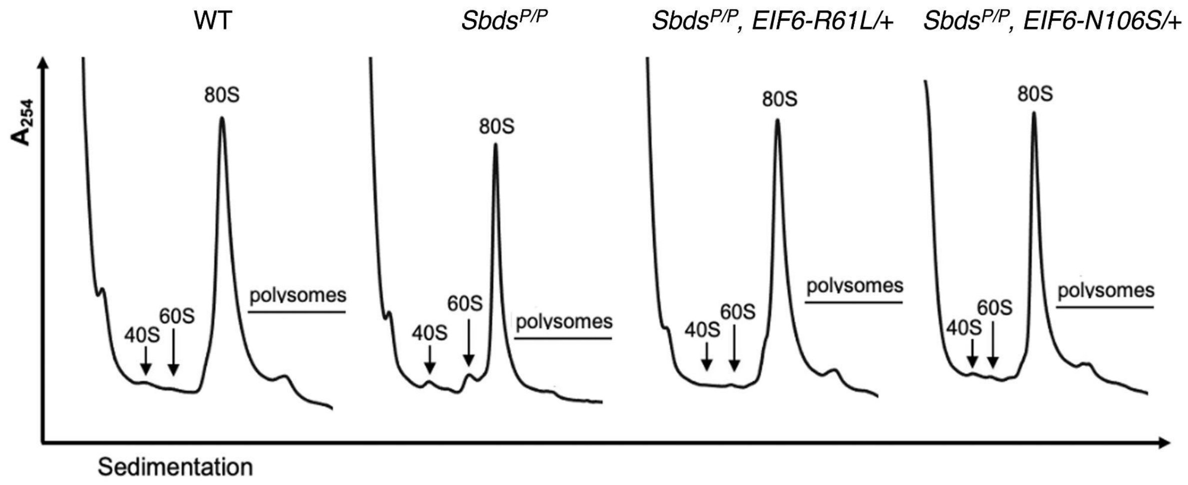

Suppl. Fig. 12. *EIF6-R61L* and *N106S* rescue the ribosome assembly defect in Sbds-deficient flies. Polysome profiles from the indicated *Drosophila* genotypes are indicated (2 replicates).

Supplementary Figure 13

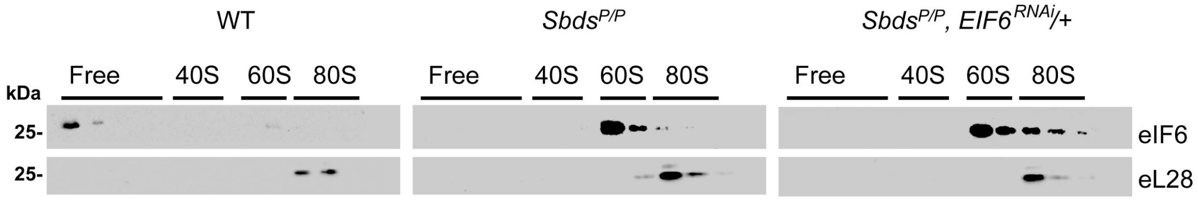

Suppl. Fig. 13. *EIF6* depletion does not increase the amount of free eIF6 in Sbds-deficient *Drosophila* cells. Cell extracts from third instar larvae were fractionated by sucrose gradient sedimentation (3 replicates) and immunoblotted to visualize the indicated proteins (2 replicates).

Supplementary Figure 14

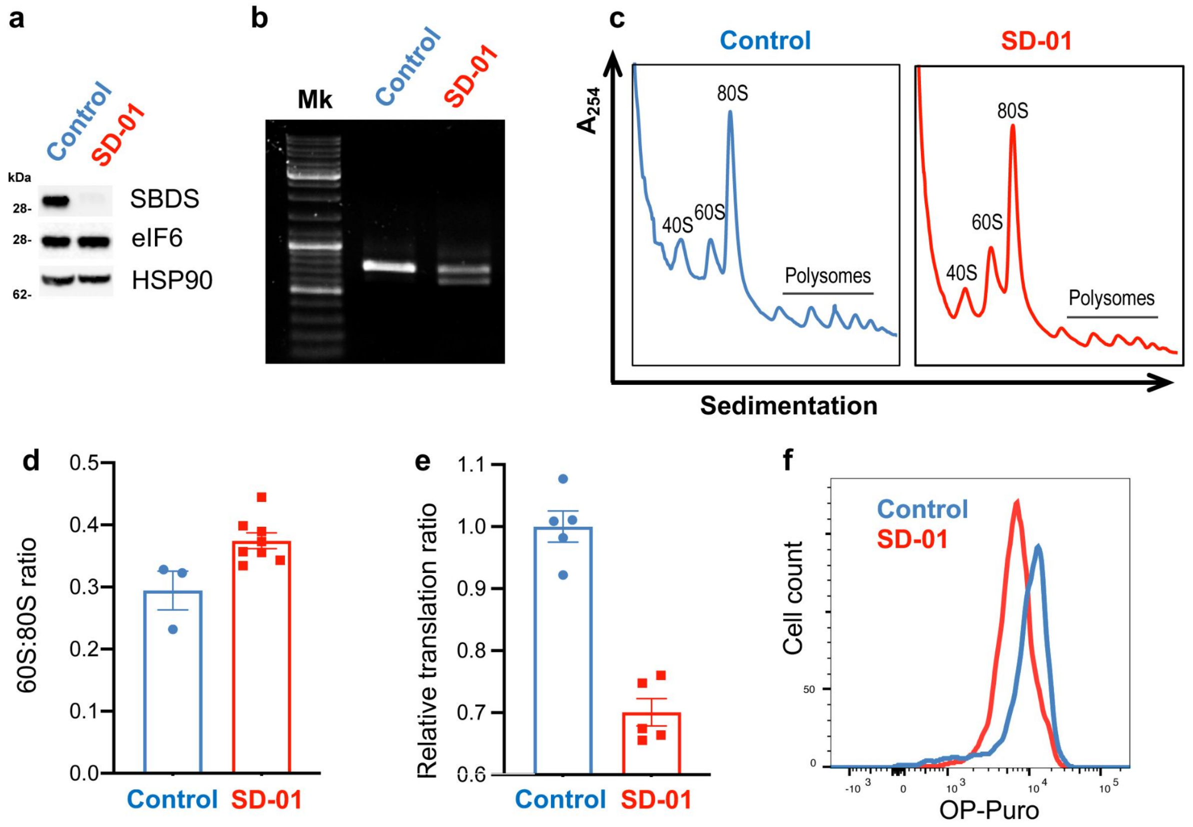

Suppl. Fig. 14. Impaired translation due to SBDS protein deficiency in SD-01 patient fibroblasts. **a** Extracts from healthy control or SD-01 patient fibroblasts were immunoblotted to visualize the indicated proteins (3 replicates). **b** RT-PCR of *SBDS* cDNA indicates a splicing anomaly in SD-01 fibroblasts, confirmed by sequencing (3 replicates). **c** Comparison of polysome profiles from control versus SD-01 patient fibroblasts. **d** Quantification of the 60S:80S ribosomal subunit ratios from polysome profiles of SD-01 (8 replicates) and control fibroblasts (3 replicates). **e, f** Relative global translation rates in fibroblasts from control (blue) versus patient SD-01 (red) (5 replicates).

Supplementary Tables

Supplementary Table S1. Frequency of eIF6 mutants in gnomAD, COSMIC and TCGA

eIF6 Variant	gnomAD: Allele count (allele frequency)	COSMIC : count (type of cancer)	TCGA : count (type of cancer)
N66H	Absent	Absent	Absent
G69S	Absent	2 (Endometrioid carcinoma - Carcinoma Adenocarcinoma)	Absent
R96W	1 (3.99e-6)	1 (Gastric adenocarcinoma)	1/11519 (stomach)
N106S	6 (2.4e-5)	Absent	1/11519 (Corpus uteri)
D112N	Absent	Absent	Absent
L133P	1 (3.98e-6)	Absent	Absent
V135M	Absent	Absent	Absent

Supplementary Table S2a. *Drosophila* genotypes

Figure	Name	Genotype
7a	$\text{Sbds}^{\text{FLAG}}$	w^{1118}*; Actin-GAL4/UAS-Sbds-FLAG*
7c	$Sbds^{RNAi}$	w^{1118}*; En-GAL4, UAS-GFP/+; UAS-*$Sbds^{RNAi}$
7d	control	w^{1118}*; Da-GAL4*
7d	$Sbds^{RNAi/}+$	w^{1118}*; Da-GAL4/UAS-* $Sbds^{RNAi}$/+
7e	control	*MS1096-GAL4/Y*
7e	$Sbds^{RNAi/}+$	*MS1096-GAL4/Y; UAS-* $Sbds^{RNAi}$/+
7g-m, S11b-g, S12, S13	WT	w^{1118}
7g-m, S11b-g, S12, S13	$Sbds^{P/P}$	w^{1118}*;* $Sbds^{P/P}$
7h, 7j	*EIF6/+*	w^{1118}*; UAS-EIF6-FLAG/+; Da-GAL4 /+*
7h, 7i, S11c	$Sbds^{P/P}$*, EIF6/+*	w^{1118}*; UAS-EIF6-FLAG/+; Da-GAL4,* $Sbds^{P}/Sbds^{P}$
7i, 7k, S11c, S11d, S12	$Sbds^{P/P}$*, EIF6-R61L/+*	w^{1118}*; UAS-EIF6-R61L-FLAG/+; Da-GAL4,* $Sbds^{P}/Sbds^{P}$
7g-m, S11c, S12	$Sbds^{P/P}$*, EIF6-N106S/+*	w^{1118}*; UAS-EIF6-N106S-FLAG/+; Da-GAL4,* $Sbds^{P}/Sbds^{P}$
7g, 7h, S11b, S13	$Sbds^{P/P}$*,* $EIF6^{RNAi}$/+	w^{1118}*; UAS-* $EIF6^{RNAi}$/+*; Da-GAL4,* $Sbds^{P}/Sbds^{P}$

S11b-d, S11f, S11g	*Sbds*$^{P/P}$, *EIF6-C56R/+*	*w*^{1118}*;UAS-EIF6-C56R-MYC/+;Da-GAL4, Sbds*^{P}*/Sbds*^{P}
7i, S11c, S11d	*Sbds*$^{P/P}$, *EIF6-R96W/+*	*w*^{1118}*;UAS-EIF6-R96W-FLAG/+;Da-GAL4, Sbds*^{P}*/Sbds*^{P}
7i, S11d	*Sbds*$^{P/P}$, *Sbds/+*	*w*^{1118}*;Actin-GAL4/UAS-Sbds-FLAG; Sbds*$^{P/P}$
7i	*Sbds*$^{P/P}$, *SBDS/+*	*w*^{1118}*;Actin-GAL4/UAS-SBDS-FLAG; Sbds*$^{P/P}$
S11d	*Sbds*$^{P/P}$, *EIF6-Y151H/+*	*w*^{1118}*;UAS-EIF6-Y151H-MYC/+;Da-GAL4, Sbds*^{P}*/Sbds*^{P}
S11d	*Sbds*$^{P/P}$ *EIF6-V192F/+*	*w*^{1118}*;UAS-EIF6-V192F-MYC/+;Da-GAL4, Sbds*^{P}*/Sbds*^{P}
7j, S11e	WT	*w*^{1118}*; Da-GAL4*
7j, S11e	*EIF6/+*	*w*^{1118}*; UAS-EIF6/+; Da-GAL4/+*
S11e	*EIF6-C56R/+*	*w*^{1118}*; UAS-EIF6-C56R/+; Da-GAL4/+*
7j, S11e	*EIF6-R61L/+*	*w*^{1118}*; UAS-EIF6-R61L/+; Da-GAL4/+*
S11e	*EIF6-R96W/+*	*w*^{1118}*; UAS-EIF6-R96W/+; Da-GAL4/+*
7j, S11e	*EIF6-N106S/+*	*w*^{1118}*; UAS-EIF6-N106S/+; Da-GAL4/+*
S11e	*EIF6-Y151H/+*	*w*^{1118}*; UAS-EIF6-Y151H/+; Da-GAL4/+*
S11e	*EIF6-V192F/+*	*w*^{1118}*; UAS-EIF6-V192F/+; Da-GAL4/+*

Supplementary Table S2b. *Drosophila* strains

Name	Genotype	Source
Wild type (WT)	w^{1118}	J. Root (University of Cambridge UK)
$Sbds^{P}$	w^{1118}; *PBac{WH}CG8549*^{f01686}*/TM6B, Tb*^{1}	Exelixis (Harvard)
$Sbds^{RNAi}$	w^{1118}; *pUAS-8549-R4*	NIG-Fly
En-GAL4	W^{1118};*P{en2.4-GAL4}e16E, P{UAS-2xEGFP}AH2*	Bloomington Drosophila Stock Center
MS1096-GAL4	w^{1118} *P{GawB-ΔKE}Bx*$^{MS1096-KE}$	Bloomington Drosophila Stock Center
$EIF6^{RNAi}$	*P{KK101259}VIE-260B*	Vienna Drosophila Resource Center
UAS-Sbds	w^{1118}; *pUAS-Sbds-FLAG*	This study
UAS-EIF6	w^{1118}; *pUAS-EIF6-FLAG*	”
UAS-EIF6-R61L	w^{1118}; *pUAS-EIF6-R61L-FLAG*	”
UAS-EIF6-R96W	w^{1118}; *pUAS-EIF6-R96W-FLAG*	”
UAS-EIF6-N106S	w^{1118}; *pUAS-EIF6-N106S-FLAG*	”
UAS-EIF6-C56R	w^{1118}; *pUAS-EIF6-C56R-MYC*	”
UAS-EIF6-Y151H	w^{1118}; *pUAS-EIF6-Y151H-MYC*	”
UAS-EIF6-V192F	w^{1118}; *pUAS-EIF6-V192F-MYC*	”
UAS-SBDS	w^{1118}; *pUAS-SBDS-FLAG*	”

Supplementary Table S3. Plasmids (Human)

Plasmid	Source
p3xFLAG-Myc-CMV-26	Sigma
p3xFLAG-*EIF6*-WT	This study
p3xFLAG-*EIF6*-N66H	This study
p3xFLAG-*EIF6*-G69S	This study
p3xFLAG-*EIF6*-R96W	This study
p3xFLAG-*EIF6*-D112N	This study
p3xFLAG-*EIF6*-L133P	This study
p3xFLAG-*EIF6*-V135M	This study

Supplementary Table S4. Oligonucleotides (Human)

Name	Sequence 5'-3'
CMV26-FLAG-NotI-eIF6-NEB-F	GGATGACGATGACAAGCTTGCGGCCGCGATGGCGGTCCGAGCTTCGTTCG
CMV26-FLAG-NotI-eIF6-NEB-R	AGAGTCGACTGGTACCGATATCAGATCTTCAGGTGAGGCTGTCAATGAGG
h-eIF6-F	GTGTGAGGGGAACCTGGGAG
h-eIF6-R	GAGCCAGGAGCCCATGGAAC
h-eIF6-N106S-F	GCTCTCAGCCTTGGGCAGTGTCACCACCTGC
h-eIF6-N106S-R	GCAGGTGGTGACACTGCCCAAGGCTGAGAGC
h-eIF6-R96W-F	TGCAGATTAGGTGGGTGGAGGAGC
h-eIF6-R96W-R	GCTCCTCCACCCACCTAATCTGCA
h-eIF6-G69S-F	GGAACAGGCACAGTCTCCTGGTACC
h-eIF6-G69S-R	GGTACCAGGAGACTGTGCCTGTTCC
h-eIF6-N66H-F	ATGTGTGTGGGGCACAGGCACGGTCTC
h-eIF6-N66H-R	GAGACCGTGCCTGTGCCCCACACACAT
h-eIF6-D112N-F	CACCACCTGCAATAACTACGTGGCCTTG
h-eIF6-D112N-R	CAAGGCCACGTAGTTATTGCAGGTGGTG
h-eIF6-L133P-F	CTGGCAGATGTGCCCAAGGTGGAAGTC
h-eIF6-L133P-R	GACTTCCACCTTGGGCACATCTGCCAG
h-eIF6-V135M-F	GATGTGCTCAAGATGGAAGTCTTCAG
h-eIF6-V135M-R	CTGAAGACTTCCATCTTGAGCACATC
h-SBDS-F	CACCATGTCGATCTTCACCCCCAC
h-SBDS-R	TTCAAATTTCTCATCTCCTTC

Supplementary Table S5. Oligonucleotides (*Dictyostelium*)

DTO1	TAAGGGCCCGATGTAAAACTATGAAAATTTTTGGATGTACTG
DTO3	CATGGATCCTATCTTTGCTCTTCAAGGCTCTAAACCATCAAAC
DTO4	AAACCGCGGAAGGCAATTAAGCCATATCATAGAATTAAAGCAC
DTO9	AAAGGGCCCATCGCAACTGTTTTCATATTGTAATCC
DTO16	CTTGTACTTGTTTAAAGGACGTACTATTG
DTO18	CATCGCACTACGTATAATTTAACACTTTGCTC
DTO28	GTATTGCAGGTACACGTACTGTTGGTCGTTTATCAG
DTO29	CTGATAAACGACCAACAGTACGTGTACCTGCAATAC
DTO30	CTCAGCATTGGGTAGTTGTATCGCCACAAATGATTATG
DTO31	CATAATCATTTGTGGCGATACAACTACCCAATGCTGAG
MR7	CATCATGAAAATTTATATTTTCAAGGTATGGCTACAAGATTACAATATGAAAACAG
MR11	AGTGCAGATCTTTATACATTATCAACAATTGAATTTC
MR15	GTAGAGACAAGTATTGCAGGTAAACGTATTGTTGG
MR16	CCAACAATACGTTTACCTGCAATACTTGTCTCTAC

Underlined residues are restriction enzyme sites.

Supplementary Table S6. Yeast strains

Yeast strain	Genotype	Source
BGY28; *sdo1^{ts}*	*MATα can1Δ::MFA1pr-HIS3 lyp1Δ his3Δ1 leu2Δ0 ura3Δ0 met15Δ0 sdo1^{ts}:kanMX*	This study
NE0257	*MATα can1Δ::MFA1pr-HIS3 lyp1Δ his3Δ1 leu2Δ0 ura3Δ0 met15Δ0 sdo1^{ts}:kanMX pRS316 URA3*	"
NE0259	*MATα can1Δ::MFA1pr-HIS3 lyp1Δ his3Δ1 leu2Δ0 ura3Δ0 met15Δ0 sdo1-VMA1-ts:kanMX pRS316 URA3 TIF6-GFP*	"
NE0261	*MATα can1Δ::MFA1pr-HIS3 lyp1Δ his3Δ1 leu2Δ0 ura3Δ0 met15Δ0 sdo1-VMA1-ts:kanMX pRS316 URA3 tif6-G14S-GFP*	"
ZBY0001	*MATα can1Δ::MFA1pr-HIS3 lyp1Δ his3Δ1 leu2Δ0 ura3Δ0 met15Δ0 sdo1-VMA1-ts:kanMX pRS316 URA3 tif6-R61L-GFP*	"
ZBY0002	*MATα can1Δ::MFA1pr-HIS3 lyp1Δ his3Δ1 leu2Δ0 ura3Δ0 met15Δ0 sdo1-VMA1-ts:kanMX pRS316 URA3 tif6-N66H-GFP*	"
ZBY0003	*MATα can1Δ::MFA1pr-HIS3 lyp1Δ his3Δ1 leu2Δ0 ura3Δ0 met15Δ0 sdo1-VMA1-ts:kanMX pRS316 URA3 tif6-N66K-GFP*	"
ZBY0004	*MATα can1Δ::MFA1pr-HIS3 lyp1Δ his3Δ1 leu2Δ0 ura3Δ0 met15Δ0 sdo1-VMA1-ts:kanMX pRS316 URA3 tif6-G69S-GFP*	"
NE0267	*MATα can1Δ::MFA1pr-HIS3 lyp1Δ his3Δ1 leu2Δ0 ura3Δ0 met15Δ0 sdo1-VMA1-ts:kanMX pRS316 URA3 tif6-R96W-GFP*	"
NE0269	*MATα can1Δ::MFA1pr-HIS3 lyp1Δ his3Δ1 leu2Δ0 ura3Δ0 met15Δ0 sdo1-VMA1-ts:kanMX pRS316 URA3 tif6-N106S-GFP*	"
NE0271	*MATα can1Δ::MFA1pr-HIS3 lyp1Δ his3Δ1 leu2Δ0 ura3Δ0 met15Δ0 sdo1-VMA1-ts:kanMX pRS316 URA3 tif6-D112E-GFP*	"
ZBY0005	*MATα can1Δ::MFA1pr-HIS3 lyp1Δ his3Δ1 leu2Δ0 ura3Δ0 met15Δ0 sdo1-VMA1-ts:kanMX pRS316 URA3*	"

	tif6-L133P-GFP	
ZBY0006	*MATα can1Δ::MFA1pr-HIS3 lyp1Δ his3Δ1 leu2Δ0 ura3Δ0 met15Δ0 sdo1-VMA1-ts:kanMX pRS316 URA3 tif6-V135M-GFP*	”
AJW3	*MATa/α lyp1Δ/+ mfα1Δ::MFα1pr-LEU2/+ can1Δ::MFA1pr-HIS3/+ his3Δ1/his3Δ1 leu2Δ0/leu2Δ0 ura3Δ0/ura3Δ0 met15Δ0/met15Δ0 sdo1Δ::NatMX4/SDO1 tif6Δ::KanMX4/TIF6*	Menne et al.[2]

Supplementary Table S7. Oligonucleotides (Yeast)

Primer	Sequence
G14S Forward	AATGAAATCAGTGTATTCTCCAAATTAACG
G14S Reverse	AGAATACACTGATTTCATTGGAGTTTTCAA
R61L Forward	ACGCGTATCATCGGTTTGATGACCGCGGGTAACC
R61L Reverse	GGTTACCCGCGGTCATCAAACCGATGATACGCGT
N66H Forward	GCGGGTCACCGTAGAGGTCTGCTAGTTC
N66H Reverse	CCTCTACGGTGACCCGCGGTCATTCTAC
N66K Forward	GCGGGTAAACGTAGAGGTCTGCTAGTTC
N66K Reverse	CCTCTACGTTTACCCGCGGTCATTCTAC
G69S Forward	ACCGTAGAAGTCTGCTAGTTCCAACCCAAA
G69S Reverse	GTTGGAACTAGCAGACTTCTACGGTTACCC
R96W Forward	AAGATTCAATGGGTAGAGGAAAGACTATC
R96W Reverse	CCTCTACCCATTGAATCTTAACGGAATCC
N106S Forward	CCTTGGGTAGCGTCATCTGTTGTAATGATTA
N106S Reverse	CAGATGACGCTACCCAAGGCAGATAGTCTTT
D112E Forward	TTGTAATGAGTACGTTGCTTTAGTGCAT
D112E Reverse	AGCAACGTACTCATTACAACAGATGACG
L133P Forward	TGATGTACCAGGCGTTGAGGTCTTCCGTC
L133P Reverse	CAACGCCTGGTACATCACTTATCAATTCT
V135M Forward	TACTAGGCATGGAGGTCTTCCGTCAAACCA
V135M Reverse	AAGACCTCCATGCCTAGTACATCACTTATC

Supplementary Table S8. Plasmids (Yeast)

Plasmid	Description	Source
pRS316	URA3 CEN	Sean Munro
pTIF6-GFP	URA3 CEN	Louise Tonkin
pTIF6-G14S-GFP	URA3 CEN	This study
pTIF6-R61L-GFP	URA3 CEN	This study
pTIF6-N66H-GFP	URA3 CEN	This study
pTIF6-N66K -GFP	URA3 CEN	This study
pTIF6-G69S-GFP	URA3 CEN	This study
pTIF6-R96W-GFP	URA3 CEN	This study
pTIF6-N106S-GFP	URA3 CEN	This study
pTIF6-D112E-GFP	URA3 CEN	This study
pTIF6-L133P-GFP	URA3 CEN	This study
pTIF6-V135M-GFP	URA3 CEN	This study

Supplementary Table S9. Oligonucleotides (*Drosophila*)

**Name**	**Sequence 5'-3'**
D-S bds-F	CACCATGTCCAAAATATTCACG
D-S bds-R	GAAGAGTTCCTCGCTCTCCAC
D-E IF6-F	CACCATGGCTCTACGCGTCC
D-E IF6-R	GGACATGTCCTCGATGAGGGC
D-E IF6-C56R-F	TGGTGCATGCGAATGTGGGCGGCCGTCGGAT
D-E IF6-C56R-R	GCTGTGCCTGGTTAAGCTTGAAGAC
D-E IF6-R61L-F	CTGCCGGATCATCGGCCTACTCACCG
D-E IF6-R96W-F	CGTGAAGATTTATTGGGTGGAGG
D-E IF6-N106S-F	CTGTCCGCGCTGGGCTCCGTTATCGC
D-E IF6-Y151H-F	CGGTGGATCCGAGACCTTCTAC
D-E IF6-Y151H-R	GGTTGCTCAGCACGGCGTGAGAGC
D-E IF6-V192F-F	CGGTGGATCCGAGACCTTCTAC
D-E IF6-V192F-R	CCATGCCGGCGGCGAGGAATTCGC
h-SBDS-F	CACCATGTCGATCTTCACCCCCAC
h-SBDS-R	TTCAAATTTCTCATCTCCTTC

Supplementary Table S10. Plasmids (*Drosophila*)

Name	Source
pTWF	The Drosophila Gateway vector collection
pPWM	The Drosophila Gateway vector collection
pRSETA-SBDS	Finch et al.[3]
pUAS-SBDS-FLAG	This study
pUAS-Sbds-FLAG	This study
pUAS-EIF6-FLAG	This study
pUAS-EIF6-R61L-FLAG	This study
pUAS-EIF6-R96W-FLAG	This study
pUAS-EIF6-N106S-FLAG	This study
pUAS-EIF6-C56R-MYC	This study
pUAS-EIF6-N106S-MYC	This study

Supplementary Table S11. Antibodies

Name	Source (RRID)	Application (dilution)
anti-α-tubulin	Sigma, #T6199 (AB_477583)	IB (1/2000), IF (1/1000)
anti-β-actin	Sigma, #A5316 (AB_476743)	IB (1/1000)
anti-cMyc	Sigma, #M5546 (AB_260581)	IB (1/500), IF (1:200)
anti-Drosophila-Sbds	A.J. Warren	IB (1/1000), IF (1/500)
anti-eIF6	GenTex, #GTX117971 (AB_11173506)	IB (1/1000)
anti-FLAG	Sigma, #F3165 (AB_259529)	IB (1/1000), IF (1/500)
Anti-DDDDK	Abcam, #ab1257 (AB_299216)	IB (1/20,000)
anti-GAPDH	Sigma, #G9545 (AB_796208)	IB (1/10,000)
anti-histone-H2B	Millipore, #07-371 (AB_310561)	IB (1/1000)
anti-histone-H3	Abcam, #ab1791 (AB_302613)	IB (1/1000)
anti-eL28	Santa Cruz, #sc-14151 (AB_2181749)	IB (1/1000)
anti-uL14	Abcam, #ab112587 (AB_10866400)	IB (1/2000)
anti-eS6	Cell signaling, #2317 (AB_2238583)	IB (1/1000)
anti-eL8	Ziemecki et al [4]	IB (1/1000)
Anti-mouse IgG HRP	Sigma, #A5278 (AB_258232)	IB (1/10,000)
anti-goat IgG HRP	Santa Cruz, #sc-2020 (AB_631728)	IB (1/10,000)
anti-rabbit IgG HRP	Cell Signaling, #7074 (AB_2099233)	IB (1/5000)
anti-mouse IgG, Alexa Fluor 488	Invitrogen, # A-21202 (AB_141607)	IF (1/1000)
anti-mouse IgG, Alexa Fluor 568	Invitrogen, # A-10037 (AB_2534013)	IF (1/1000)
anti-rabbit IgG, Alexa Fluor 488	Invitrogen, # A-21206 (AB_2535792)	IF (1/1000)

anti-rabbit IgG, Alexa Fluor 568	Invitrogen, # A-10042 (AB_2534017)	IF (1/500)
anti-rabbit IgG, Alexa Fluor 647	Invitrogen, # A-21244 (AB_2535812)	IF (1/500)

Supplementary References

1. Gouet, P., Courcelle, E., Stuart, D.I. & Metoz, F. ESPript: analysis of multiple sequence alignments in PostScript. *Bioinformatics* **15**, 305-8 (1999).

2. Menne, T.F. *et al.* The Shwachman-Bodian-Diamond syndrome protein mediates translational activation of ribosomes in yeast. *Nat Genet* **39**, 486-495 (2007).

3. Finch, A.J. *et al.* Uncoupling of GTP hydrolysis from eIF6 release on the ribosome causes Shwachman-Diamond syndrome. *Genes Dev* **25**, 917-929 (2011).

4. Ziemiecki, A., Muller, R.G., Fu, X.C., Hynes, N.E. & Kozma, S. Oncogenic activation of the human trk proto-oncogene by recombination with the ribosomal large subunit protein L7a. *EMBO J* **9**, 191-6 (1990).
