# Supplementary material for: Somatic genetic rescue of a germline ribosome assembly defect
Source: Nat Commun. 2021 Aug 19;12:5044. doi: 10.1038/s41467-021-24999-5 (PMC8377010; doi:10.1038/s41467-021-24999-5)
Supplement: Supplementary file 5 — Description of additional supplementary files [file 41467_2021_24999_MOESM5_ESM.docx]

Description of additional supplementary files

Title: Supplementary Data 1

Description: List of SDS patients (Excel file).

Title: Supplementary Data 2

Description: CADD scores of all *EIF6* SNVs (Excel file).

Title: Supplementary Data 3

Description: SNPs /BAF (Excel file).
