# Supplementary material for: Somatic genetic rescue of a germline ribosome assembly defect
Source: Nat Commun. 2021 Aug 19;12:5044. doi: 10.1038/s41467-021-24999-5 (PMC8377010; doi:10.1038/s41467-021-24999-5)
Supplement: Supplementary file 6 — Reporting Summary [file 41467_2021_24999_MOESM6_ESM.pdf]

nature research

Corresponding author(s): Patrick Revy & Alan J. Warren

Last updated by author(s): 2021 05 11

Reporting Summary

Nature Research wishes to improve the reproducibility of the work that we publish. This form provides structure for consistency and transparency in reporting. For further information on Nature Research policies, see our Editorial Policies and the Editorial Policy Checklist.

Statistics

For all statistical analyses, confirm that the following items are present in the figure legend, table legend, main text, or Methods section.

n/a	Confirmed	
☐	☒	The exact sample size (*n*) for each experimental group/condition, given as a discrete number and unit of measurement
☐	☒	A statement on whether measurements were taken from distinct samples or whether the same sample was measured repeatedly
☐	☒	The statistical test(s) used AND whether they are one- or two-sided<br>*Only common tests should be described solely by name; describe more complex techniques in the Methods section.*
☒	☐	A description of all covariates tested
☒	☐	A description of any assumptions or corrections, such as tests of normality and adjustment for multiple comparisons
☐	☒	A full description of the statistical parameters including central tendency (e.g. means) or other basic estimates (e.g. regression coefficient) AND variation (e.g. standard deviation) or associated estimates of uncertainty (e.g. confidence intervals)
☐	☒	For null hypothesis testing, the test statistic (e.g. *F*, *t*, *r*) with confidence intervals, effect sizes, degrees of freedom and *P* value noted<br>*Give P values as exact values whenever suitable.*
☒	☐	For Bayesian analysis, information on the choice of priors and Markov chain Monte Carlo settings
☒	☐	For hierarchical and complex designs, identification of the appropriate level for tests and full reporting of outcomes
☐	☒	Estimates of effect sizes (e.g. Cohen's *d*, Pearson's *r*), indicating how they were calculated

Our web collection on statistics for biologists contains articles on many of the points above.

Software and code

Policy information about availability of computer code

Data collection	See Supplementary Methods File
Data analysis	VMD 1.9.3; GROMACS 2019.6; Grace plotting tool v5.1.25; GNU Image Manipulation Program v2.10.24; Image J v10.4; Photoshop CS5; Image Lab (Bio-Rad) v6.0.1; FlowJo v10.7; Prism 9.1.2

For manuscripts utilizing custom algorithms or software that are central to the research but not yet described in published literature, software must be made available to editors and reviewers. We strongly encourage code deposition in a community repository (e.g. GitHub). See the Nature Research guidelines for submitting code & software for further information.

Data

Policy information about availability of data

All manuscripts must include a data availability statement. This statement should provide the following information, where applicable:

- Accession codes, unique identifiers, or web links for publicly available datasets
- A list of figures that have associated raw data
- A description of any restrictions on data availability

Authors can confirm that all relevant data are included in the paper and/or its supplementary information files.

Field-specific reporting

Please select the one below that is the best fit for your research. If you are not sure, read the appropriate sections before making your selection.

☒ Life sciences ☐ Behavioural & social sciences ☐ Ecological, evolutionary & environmental sciences

For a reference copy of the document with all sections, see nature.com/documents/nr-reporting-summary-flat.pdf

Life sciences study design

All studies must disclose on these points even when the disclosure is negative.

Sample size	For each Drosophila experiment, at least 3 replicates were performed and the sample size contained a minimum of 156 flies. These numbers were chosen to provide adequate power.
Data exclusions	No data were excluded from the analysis
Replication	Reported results were consistently replicated across multiple experiments, with all replicates generating similar results
Randomization	Flies of the same genotype were randomly allocated to experimental group
Blinding	Experimenters were not blinded to fly genotypes as the phenotypes would be clear to any observer.

Reporting for specific materials, systems and methods

We require information from authors about some types of materials, experimental systems and methods used in many studies. Here, indicate whether each material, system or method listed is relevant to your study. If you are not sure if a list item applies to your research, read the appropriate section before selecting a response.

Materials & experimental systems

n/a	Involved in the study	
☐	☒	Antibodies
☐	☒	Eukaryotic cell lines
☒	☐	Palaeontology and archaeology
☐	☒	Animals and other organisms
☐	☒	Human research participants
☒	☐	Clinical data
☒	☐	Dual use research of concern

Methods

n/a	Involved in the study	
☒	☐	ChIP-seq
☐	☒	Flow cytometry
☒	☐	MRI-based neuroimaging

Antibodies

Antibodies used	Listed in Supplementary Table S11.
Validation	The anti Drosophila Sbds antibody is validated in Figure 7. All other antibodies are commercially available and validated by the manufacturer.

Eukaryotic cell lines

Policy information about cell lines

Cell line source(s)	Yeast strains and sources are listed in Supplementary Table S6. BGY28, NE0257, NE0259, NE0261, ZBY0001, ZBY0002, ZBY0003, ZBY0004, NE0267, NE0269, NE0271, ZBY0005, ZBY0006, AJW3 were all generated in my lab and are available on request.<br>Dictyostelium cell line Ax2 is available through Dictybase (http://dictybase.org/), strain ID DBS0235521.<br>HEK293T cell line was obtained from ATCC.
Authentication	Cell lines were authenticated using PCR and genomic sequencing
Mycoplasma contamination	All cell lines were negative as inferred by negative PCR results.
Commonly misidentified lines (See ICLAC register)	No commonly misidentified cell lines were used in this study

Animals and other organisms

Policy information about studies involving animals; ARRIVE guidelines recommended for reporting animal research

Laboratory animals	We used Drosophila melanogaster strains listed in Supplemntary Table S2a.<br>w1118, gift from J. Root (University of Cambridge UK); w1118; PBac{WH}CG8549f01686/TM6B, Tb1, Exelixis (Harvard); w1118; pUAS-8549-R4, NIG-Fly; W1118;P{en2.4-GAL4}e16E, P{UAS-2xEGFP}AH2, Bloomington Drosophila Stock Center; w1118 P{GawB-ΔKE}BxMS1096-KE, Bloomington Drosophila Stock Center; P{KK101259}VIE-260B, Vienna Drosophila Resource Center.<br>Either adult flies or larvae were used as specified in the Methods and Figure legends.
Wild animals	This study did not involve wild animals
Field-collected samples	This study did not involve samples collected in the field.
Ethics oversight	Ethics approval was not required for experiments on invertebrates

Note that full information on the approval of the study protocol must also be provided in the manuscript.

Human research participants

Policy information about studies involving human research participants

Population characteristics	Blood and/or bone marrow samples were obtained from patients with Shwachman-Diamond syndrome (Age and gender are listed in Supplementary data 1)
Recruitment	Samples were collected from consenting patients attending hospital out-patient clinics
Ethics oversight	Informed and written consent was obtained from donors and patients. The study and protocols comply with the 1975 Declaration of Helsinki as well as with the local legislation and ethical guidelines from the Comité de Protection des Personnes de l'Ile de France II and the French advisory committee on data processing in medical research. The INSERM Institutional Review Board also approved this study.

Note that full information on the approval of the study protocol must also be provided in the manuscript.

Flow Cytometry

Plots

Confirm that:

☒ The axis labels state the marker and fluorochrome used (e.g. CD4-FITC).

☒ The axis scales are clearly visible. Include numbers along axes only for bottom left plot of group (a 'group' is an analysis of identical markers).

☒ All plots are contour plots with outliers or pseudocolor plots.

☒ A numerical value for number of cells or percentage (with statistics) is provided.

Methodology

Sample preparation	The methodology is described in detail in the methods section of the paper
Instrument	FACScalibur.
Software	FlowJo
Cell population abundance	The percentage are indicated in the Supplementary figure Fig.5
Gating strategy	FSC/SSC gates were used to analyse living cells.

☒ Tick this box to confirm that a figure exemplifying the gating strategy is provided in the Supplementary Information.
